# Evaluating the evidence for biotypes of depression: Methodological replication and extension of Drysdale et al. (2017)

**DOI:** 10.1016/j.nicl.2019.101796

**Published:** 2019-03-27

**Authors:** Richard Dinga, Lianne Schmaal, Brenda W.J.H. Penninx, Marie Jose van Tol, Dick J. Veltman, Laura van Velzen, Maarten Mennes, Nic J.A. van der Wee, Andre F. Marquand

**Affiliations:** a Department of Psychiatry, Amsterdam UMC, Amsterdam, the Netherlands; b Orygen, The National Centre of Excellence in Youth Mental Health, Parkville, Australia; c Centre for Youth Mental Health, The University of Melbourne, Melbourne, Australia; d Cognitive Neuroscience Center, University Medical Center Groningen, University of Groningen, Groningen, the Netherlands; e Donders Institute for Brain, Cognition and Behaviour, Radboud University, Nijmegen, the Netherlands; f Department of Psychiatry, Leiden University Medical Center, Leiden, the Netherlands

**Keywords:** Clustering, Anxiety, Major depressive disorder, Machine learning, Replication

## Abstract

**Background:**

Psychiatric disorders are highly heterogeneous, defined based on symptoms with little connection to potential underlying biological mechanisms. A possible approach to dissect biological heterogeneity is to look for biologically meaningful subtypes. A recent study Drysdale et al. (2017) showed promising results along this line by simultaneously using resting state fMRI and clinical data and identified four distinct subtypes of depression with different clinical profiles and abnormal resting state fMRI connectivity. These subtypes were predictive of treatment response to transcranial magnetic stimulation therapy.

**Objective:**

Here, we attempted to replicate the procedure followed in the Drysdale et al. study and their findings in a different clinical population and a more heterogeneous sample of 187 participants with depression and anxiety. We aimed to answer the following questions: 1) Using the same procedure, can we find a statistically significant and reliable relationship between brain connectivity and clinical symptoms? 2) Is the observed relationship similar to the one found in the original study? 3) Can we identify distinct and reliable subtypes? 4) Do they have similar clinical profiles as the subtypes identified in the original study?

**Methods:**

We followed the original procedure as closely as possible, including a canonical correlation analysis to find a low dimensional representation of clinically relevant resting state fMRI features, followed by hierarchical clustering to identify subtypes. We extended the original procedure using additional statistical tests, to test the statistical significance of the relationship between resting state fMRI and clinical data, and the existence of distinct subtypes. Furthermore, we examined the stability of the whole procedure using resampling.

**Results and conclusion:**

As in the original study, we found extremely high canonical correlations between functional connectivity and clinical symptoms, and an optimal three-cluster solution. However, neither canonical correlations nor clusters were statistically significant. On the basis of our extensive evaluations of the analysis methodology used and within the limits of comparison of our sample relative to the sample used in Drysdale et al., we argue that the evidence for the existence of the distinct resting state connectivity-based subtypes of depression should be interpreted with caution.

## Highlights

• Using methods of Drysdale et al. (2017) we have obtained extremely high canonical correlations and optimal 3 cluster solution
• These, however, were not statistically significant after performing sufficiently rigorous statistical tests
• We argue that the methods used in Drysdale et al. (2017) do not provide convincing evidence for biotypes of depression

## 1 Introduction

Psychiatric disorders are highly heterogeneous in terms of symptom presentation and underlying biological mechanisms and are diagnosed exclusively in terms of symptoms, which may not correspond to biological causes (Kapur et al., 2012) This, together with frequent comorbidities between disorders, complicates clinical diagnosis and hinders efforts to understand biological mechanisms of disorders and to develop better treatments. This problem has been known for a long time, but little progress has been made and clinical decision-making is still mostly done on the basis of symptoms. Recent initiatives such as the Research Domain Criteria (RDoC) (Insel, 2014) aim to address this issue of heterogeneity by going beyond current diagnostic categories and focusing analysis on different domains of functioning and pathology across multiple levels of analysis, including clinical symptoms, behavior, and biology.

Many studies have used data-driven clustering methods in order to find new subgroups of clinical populations, based on either clinical or biological data, with some degree of success (Marquand et al., 2016; Chekroud et al., 2017). The dominant approach of clustering based on clinical symptoms alone can provide new insights into psychopathology, however, it may not yield subtypes that reflect underlying biological differences. On the other hand, the variability of biological data is more often than not unrelated to any specific psychiatric disorder or symptom class. Thus, clustering based on biological data alone may detect subtypes that are unrelated to psychiatric pathologies, and instead reflect dominant nuisance variance in the data such as groups of people with similar brain size or body type or common ancestry in the case of genetics. One way to overcome these limitations is to constrain the search for subtypes in biological data to lie along axes of variance that are related to psychiatric symptomatology. However, few studies have used such an approach (Marquand et al., 2016).

A prominent example following this approach is a recent study by Drysdale an colleagues (Drysdale et al., 2017) that aimed to stratify major depressive disorder (MDD) on the basis of biology and behavior and suggested the existence of four distinct biotypes. The authors used canonical correlation analysis (CCA)(Hotelling, 1936) to identify a two-dimensional mapping between functional connectivity measures derived from resting state fMRI (RS-fMRI) data and MDD symptoms. CCA is a well-established method for finding multivariate associations between different data sources and has been used extensively in clinical neuroimaging, for example for finding associations between neuroimaging data and behavior (Marquand et al., 2017; Smith et al., 2015) and neuroimaging and genetics (Le Floch et al., 2012). Next, Drysdale et al. applied a hierarchical clustering on two components derived from CCA and identified four different clusters of MDD patients, i.e. the aforementioned biotypes. Impressively, these biotypes were predictive of transcranial magnetic stimulation (TMS) treatment response, and they were also evaluated in an independent sample. However, the study has some methodological limitations. For example, the existence of distinct clusters was not conclusively established in that the authors did not test the possibility that subjects were sampled from a single continuous distribution without underlying clusters. Nevertheless, the results are promising, and if replicated it would be an important step towards understanding biological mechanisms of MDD.

The aim of this study is to apply the analysis methods used by Drysdale et al. to a completely independent sample from a different clinical population of patients with depression and anxiety disorders, namely data from the Netherlands Study of Depression and Anxiety (NESDA) (Penninx et al., 2008) and Mood Treatment with Antidepressants or Running (MOTAR) study (Lever-van Milligen et al., in preparation). These studies together create a relatively large cohort containing a heterogeneous sample of subjects with depression, anxiety and comorbid depression and anxiety, thus capturing a wider range of possible clinical and biological profiles relative to the study by Drysdale and colleagues, which included mainly hospitalized treatment-resistant patients in a currently active depressive episode. The original study used 220 patients as a cluster discovery dataset and an additional 92 patients from the same cohort as a replication dataset. Our combined dataset of NESDA and MOTAR includes a cohort of 187 participants with clinical measures measured by a comparable clinical instrument. Specifically, using the same procedure as in (Drysdale et al., 2017) but in a different clinical population, we aimed to answer the following questions: 1) can we find a statistically significant and reliable relationship between brain connectivity and clinical symptoms? 2) Is the identified relationship similar to the one found in the original study? 3) Can we identify distinct and reliable subtypes? 4) If so, do they have similar clinical profiles as the subtypes identified in the original study?

We consider that the type of methodological replication we have performed, that is, in a different depressed population and with different measures of depressive symptoms provides a strong test of the subtypes found in Drysdale et al. (2017) in that if our findings were supportive, this replication would provide strong evidence for external validity of the original findings. However, if they do not support the original findings it could be difficult to determine whether that is due to the unreliability of the original findings or differences in the replication sample. In addition and in view of the methodological considerations described above, we will perform a critical evaluation of methods used by Drysdale et al. and provide a recommendation for future studies. We will argue that in the original study, whilst there is evidence for the relationship between resting state connectivity and clinical symptoms, the presented evidence does not conclusively support the existence of biotypes of depression as proposed in the original study.

## 2 Methods

**Fig. 1:**
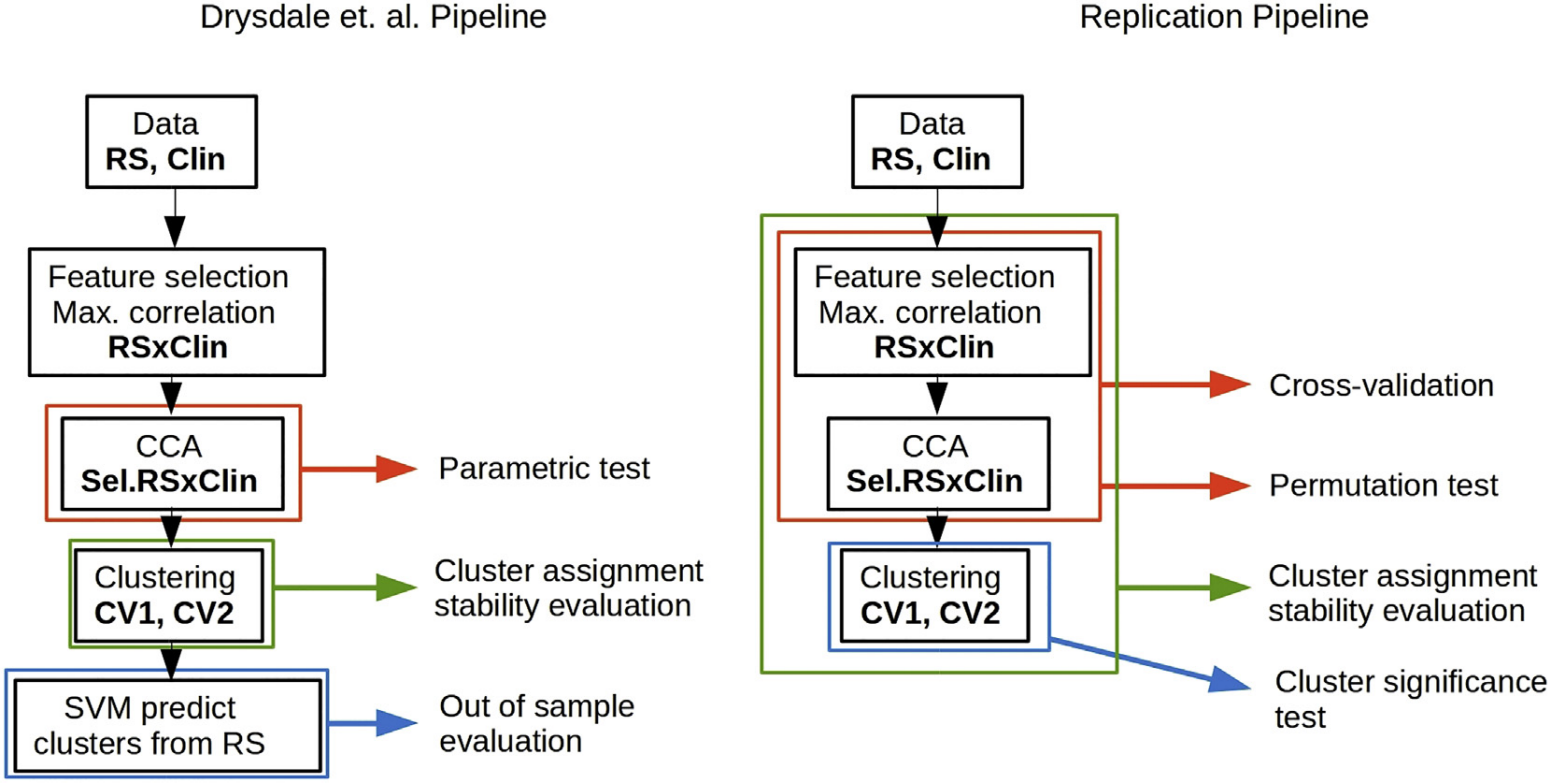
A scheme of the pipeline used in the original study and our pipeline. Data: in the original study, 220 depressed subjects have been analyzed as a part of a “cluster discovery” set and an additional 92 subjects were used as evaluation set. The clinical data (**Clin**) consisted of 17 HAM-D items. We have used 187 subjects with depression, anxiety disorder or depression-anxiety comorbidity. The clinical data consisted of 17 IDS items that best-matched the HAM-D item used in the original study. After preprocessing of fMRI data (**RS**), a correlation matrix between selected regions was created, resulting in ~35,000 features. A small subset of features (178 in the original study and 150 in our study) were selected based on their correlation with clinical symptoms (**Sel.RS**). Then, CCA was performed using these selected features and clinical symptoms. In the original study, a parametric test was used to the established statistical significance of CCA without taking a previous feature selection into an account. Hierarchical clustering was performed on first two resting state connectivity canonical variates (**CV1, CV2**). We have included an additional test, to test if the data cluster more than what is expected from data sampled from a Gaussian distribution. Stability of cluster assignment was evaluated in the original study by resampling of CV1 and CV2, We have extended the resampling stability evaluation to feature selection (in addition to the CCA procedures). **Out of sample evaluation**: in the original study, an additional 92 subjects were assigned to clusters according to a SVM model and clinical profiles of these clusters were compared to clinical profiles of clusters obtained in the cluster discovery set. We have evaluated the reproducibility of canonical correlations directly, using 10-fold cross-validation.

We conducted our analysis as closely to possible and to our best understanding of the published analysis pipeline in the Drysdale et al. study. Several details related to the analysis were not specified in the original paper and were clarified via personal communication with the corresponding author. We included several additional validation steps for CCA and cluster analysis. Our aim was to replicate the analysis steps related to the creation and evaluation of subtypes, we did not try to replicate additional analyses performed in the original study such as classification of healthy and depressed subjects or the prediction of TMS treatment response. We describe our pipeline and the Drysdale et al. pipeline below and provide a side by side schematic comparison in Fig. 1.

### 2.1 Sample characteristics

All our analyses were performed on 187 subjects from NESDA and MOTAR samples diagnosed according to DSM-IV criteria with MDD, an anxiety disorder (i.e. panic disorder, social phobia or generalized anxiety disorder) or both MDD and an anxiety disorder, established using the structured Composite International Diagnostic Interview (CIDI, version 2.1) (Robins et al., 1988) at the time of intake. At the time of scanning, subjects were additionally evaluated using the Inventory of Depressive Symptomatology (IDS) (Rush et al., 1986) and the Beck Anxiety Inventory (BAI). See Table 2 (Beck et al., 1988). At the time of scanning, 18% of subjects were in remission for both IDS and BAI using common clinical cutoffs. Considering IDS cutoffs alone, 30% of subjects were not actively depressed at the time of scanning and an additional 34% of subjects had only mild severity.

The original Drysdale et al. study included 220 subjects in a cluster discovery set and an additional 92 subjects in a validation set with an active episode of MDD and a history of treatment resistance. In our sample, 151 subjects came from the baseline assessment of NESDA (Penninx et al., 2008), which is large naturalistic cohort study of depression and anxiety (here, we have included the complete NESDA fMRI substudy). Additional descriptions of the NESDA cohort can be found in (van Tol et al., 2011). Clinical characteristics stratified by study site are in Table 2. An additional 36 subjects were from the baseline assessment of the MOTAR study (Lever-van Milligen et al., in preparation), which is a randomized controlled treatment study (antidepressants or running therapy).

Inclusion criteria for MOTAR and NESDA patient samples included current major depressive disorder or current anxiety disorder (social phobia, generalized anxiety disorder or panic disorder). Exclusion criteria included use of antidepressants or other psychotropic medication except benzodiazepine, presence of other psychiatric disorder except depressive or anxiety disorder (bipolar disorder, psychosis, addictive disorder), presence of acute suicidal risk, major neurological or internal disorder, pregnancy or other known contra-indications for MRI such as presence of metal objects or claustrophobia. MOTAR had additional exclusion criteria: participation in regular exercise (at least once a week) and medical contra-indications to running therapy or antidepressant use confirmed by a physician.

In addition to the main analyses we report below, we also repeated the analyses excluding patients with a ‘pure’ anxiety diagnosis, since these were not included in the cluster discovery sample of the original Drysdale study. These analyses are reported in the supplementary material and led to similar conclusions.

All participants gave written informed consent. Studies were approved by the Central Ethics Committees of the participating medical centers: Leiden University Medical Center (LUMC), Amsterdam Medical Center (AMC), and University Medical Center Groningen (UMCG) for NESDA and Ethics committee of Amsterdam Medical Center (AMC) for MOTAR.

### 2.2 Resting-state fMRI

Participants from NESDA were scanned at one of the three participating scan centers and at one scan center for the MOTAR study. All imaging data were acquired on a Philips 3.0-T Achieva MRI scanner. A sense-8 (UMCG and LUMC) and a sense-6 (AMC) channel head coil was used for radio frequency transmission and reception for the NESDA study and Philips 3 T Achieva with 32 and 8 channel receive head coils were used for the MOTAR study. RS-fMRI data were acquired using T2*-weighted gradient-echo echo-planar imaging with the following scan parameters for the NESDA sample: Amsterdam and Leiden centers: 200 whole-brain volumes; repetition time (TR) = 2300 ms; echo time (TE) = 30 ms; flip angle = 80°; 35 axial slices; no slice gap; FOV = 220 × 220 mm; in plane voxel resolution = 2.3 mm × 2.3 mm; slice thickness = 3 mm; same in Groningen, except: TE = 28 ms; 39 axial slices; in plane voxel resolution = 3.45 mm × 3.45 mm. And for the MOTAR sample: 210 whole-brain volumes; repetition time (TR) = 2300 ms; echo time (TE) = 28 ms, flip angle = 76.1°, 37 axial slices, no slice gap; FOV = 240 × 240, in plane voxel resolution = 3.3 mm × 3.3 mm using interleaved slice acquisition order. T1-weighted image was acquired with the repetition time (TR) = 9 ms; echo time (TE) = 3.5 ms; flip angle = 8°; 170 sagittal slices; no slice gap; FOV = 256 × 256 mm; in plane voxel resolution = 1 mm × 1 mm; slice thickness = 1 mm.

Preprocessing steps in the original study were performed using Analysis of Functional Neuroimages (AFNI) software package, using motion scrubbing and ANATICOR to deal with artifacts. Here we have performed preprocessing using FSL 5.0.8. Steps included motion correction using McFLIRT(Jenkinson et al., 2002) using default parameters and registering each volume to the middle time point, grand mean scaling of the fMRI time series, spatial smoothing with 6 mm Gaussian kernel, motion artifacts removal using ICA-AROMA (Pruim et al., 2014). Additional nuisance signal regression compliant with the pipeline recommended in (Pruim et al., 2014) was performed by regressing out mean signals from cerebrospinal fluid (CSF) and white matter extracted using participant-level masks obtained by multiplying participant-level CSF and white matter segmentations obtained by automated segmentation using FSL's FAST (Zhang et al., 2001) with the MNI152-based CSF and white matter masks provided as part of FSL and thresholded with a 0.95 threshold. The data were then high pass filtered with a cut-off frequency of 0.01 Hz. As in the original study, no slice timing correction was performed. The resulting RS-fMRI images were registered to Montreal Neurological Institute (MNI) space using transformation matrices obtained from the first co-registration of functional images to T1 image using the boundary based registration tool and registering the T1 images to MNI template brain using FMRIB's linear image registration tool (FLIRT) (Jenkinson et al., 2002). Following preprocessing, we performed carefully assessed data quality in alignment with the checks performed in the original study. These are reported in the supplementary material and show that the level of head motion in our cohort was similar to that in the Drysdale study. Note that there are some differences between the pipeline we employed here and that was used in the Drysdale study. Most notably, we employed ICA-AROMA in place of volume censoring. There is good evidence that ICA-AROMA does at least as well as volume censoring in removing motion artifacts, whilst preserving greater temporal degrees of freedom in the fMRI time series (Pruim et al., 2014; Parkes et al., 2018). We show that ICA-AROMA performed well in removing head motion-related artifacts in our data (Supplementary fig. 4).

Next, correlation matrices were created using 264 cortical parcellations proposed by Power et al. (2011) plus an additional 13 regions, including the left and right caudate, amygdala, hippocampus, nucleus accumbens and subgenual anterior cingulate cortex, as in the original Drysdale et al. study. We averaged all voxels within each region to create a single time series per region. We have discarded 1% of regions with the lowest signal to noise ratio, and subsequently created a correlation matrix by computing pairwise Pearson's correlation coefficients between all regions. This resulted in 38,000 connectivity features (lower diagonal of 277*277 correlation matrix), which were later reduced to 37,675 by discarding regions with insufficient coverage in >10% of subjects. These correlations were transformed using Fisher's z-transform and linear effects of age, frame-wise displacement and dummy coded study site were regressed out.

### 2.3 Clinical characteristics

The original study used depressive symptom scores of the Hamilton rating scale for depression (HAMD) (Hamilton, 1960) in their analyses. Here we used depressive symptom scores derived from the self-rated Inventory of Depressive Symptomatology (IDS) (Rush et al., 1986). This inventory was developed as an improvement over HAMD, aiming to improve the coverage of common MDD symptoms (Rush et al., 1996). However, to make our study comparable to the original study, we used only a subset of 17 IDS items that best matched the items of the HAMD (Table 1).

**Table 1:** HAM-D items used in the original study and best-matched IDS items used in this study.

HAMD item	IDS item
Mood	Feeling sad
Guilt	Self criticism and blame
Suicide	Thoughts of death or suicide
Early insomnia	Early insomnia
Mid insomnia	Mid insomnia
Late insomnia	Late insomnia
Anhedonia	Capacity for pleasure or enjoyment (excluding sex)
Retardation	Psychomotor retardation
Agitation	Psychomotor agitation
Anxiety psychological	Feeling anxious or tense
Anxiety physiological	Other bodily symptoms/sympathetic arousal
Somatic gastro-internal	Gastrointestinal complaints
Fatigue/aches/low energy	Energy level/fatiguability
Genital	Interest in sex
Hypochondria	Somatic complains
Weight loss	Weight loss
Insight	Sensitivity

### 2.4 Feature selection

From the 37,675 connectivity features (the lower diagonal of the functional correlation matrix), we selected the subset of features with the highest Spearman's correlation with any of the 17 IDS symptoms. In the original study, according to communication with the corresponding author, during feature selection, the number of RS-fMRI features corresponding to approximately 80% of the total number of participants were retained, which corresponds to 176 RS-fMRI connectivity features in a sample of 220 participants in the original study. Here we selected the top 150 RS-fMRI features with the highest Spearman's correlation with any of the 17 IDS symptoms to preserve the same feature to subjects ratio (80% of 187 subjects).

### 2.5 Canonical correlation analysis

Next, following the original study, we performed a canonical correlation analysis (CCA) on the selected RS-fMRI connectivity features and depressive symptoms. Canonical correlation analysis (Hotelling, 1936) is a multivariate statistical method that seeks an association between two sets of variables. CCA is the most general multivariate technique with multiple regression, MANOVA, and discriminant analysis all as special cases of CCA (multiple regression is a CCA with only one variable in Y, MANOVA, and discriminant analysis are CCA with binary variables in X or Y). Given the two multidimensional datasets X (e.g. clinical features) and Y (e.g. RS-fMRI connectivity features), canonical correlation analysis finds a linear combination of X that maximally correlates with a linear combination of Y. This linear combinations of X and Y are new variables, called canonical variates. Both canonical variates for X and Y are called a canonical pair and the correlation between canonical variates is called a canonical correlation. Multiple canonical pairs can be found with a constraint that each subsequent canonical pair has to be uncorrelated with all the previous ones.

CCA is also closely related to PCA with a difference that CCA performs eigen decomposition of the cross-correlation matrix instead of the correlation matrix. In PCA the first principal component explains the largest amount of variance in the data, and each subsequent principal component explains a (smaller) maximal amount of variance that is orthogonal to all the previous ones. In CCA the first canonical variate of X explains the largest amount of variance in Y and each subsequent canonical variate is explaining less of variance in Y and is orthogonal to all the previous canonical variates. In more detail, the squared canonical correlations are eigenvalues of the matrix:

$$R={R_{\mathrm{yy}}}^{-1}R_{\mathrm{yx}}{R_{\mathrm{xx}}}^{-1}R_{\mathrm{xy}}$$

where **R**_xx_ and **R**_yy_ are correlation matrices of X and Y, respectively, and **R**_xy_ and **R**_yx_ are cross-correlation matrices of variables from X with variables from Y. Coefficients that create the canonical variates are the respective eigenvectors of R. CCA can be also thought of as a dimensionality reduction step, where the original data of X and Y are mapped into a lower dimensional space of canonical variates whose dimensions are highly correlated between datasets X and Y, in our case between RS-fMRI connectivity measures and clinical symptoms.

#### 2.5.1 Permutation test

Traditionally, the significance of canonical correlations is established using a Wilk's lambda statistic and this was also used in the Drysdale study (Conor Liston, personal communication). This statistic has an approximately *chi-square* null distribution with *pq* degrees of freedom, *p* and *q* being the number of variables in X and Y. This significance test, however, does not take into account the feature pre-selection step that selected RS-fMRI connectivity features most correlated with clinical symptoms. As this pre-selection step was done in the same dataset as the CCA was performed on and tested, this likely results in too optimistic *p*-values. To avoid overly optimistic p-values, we performed a permutation test of the whole procedure, i.e. feature selection followed by CCA. The whole feature selection and CCA cycle was repeated for each permutation with the rows of clinical symptoms shuffled so that they no longer corresponded to rows of RS-fMRI connectivity features. We performed 1999 permutations, which created a null distribution of canonical correlations and estimated the Wilk's lambda statistic. The interpretation of Wilk's lambda statistic is also important, because it does not describe the significance of a single component in isolation. Instead, the first canonical correlation is defined as det(E)/det(E + H), where E is the error sum of squares and cross products matrix and H is model sum of squares and cross products matrix. Its significance should be interpreted as the significance of the whole decomposition, not the first component. The significance of the Wilk's lambda statistic for the second canonical correlation is interpreted as the significance of the whole decomposition after removing the variance accounted for by the first canonical correlation and so forth. In addition, if the canonical correlation from a given model order (e.g. first canonical correlation) is not significant, all correlations of a lower order (e.g. second onwards) should not be taken to be significant either, even if one or more of the derived *p*-values show nominal significance (Sherry and Henson, 1981).

#### 2.5.2 Cross-validation

CCA is prone to overfitting and although canonical correlations may seem high and even be statistically significant, they are often much lower in an independent dataset (see e.g. (Le Floch et al., 2012)). This might give an impression that the found association between modalities (RS-fMRI connectivity measures and clinical symptoms) is much stronger than it would be in an independent hold-out dataset. In the original study, the canonical correlation in the independent data set was not evaluated directly in an independent dataset, but rather the authors relied on the derived biotypes to have similar symptom profiles in the independent evaluation dataset.

Here we chose to estimate the magnitude of canonical correlation out of the training sample, using stratified 10-fold cross-validation. The dataset was divided into ten subsets with an approximately constant number of subjects from each study site across all subsets. Nine subsets were used as a training set and the remaining subset as a test set. A feature selection procedure, as described above, was performed using subjects from the training set only. In the test set, canonical variates and their respective canonical correlations were created using coefficients from the CCA performed in the training set.

#### 2.5.3 Stability of canonical loadings

Since CCA frequently yields unstable solutions, we also examined the stability of canonical loadings (i.e. structure coefficients, a univariate correlation between a variable and canonical variate) (Sherry and Henson, 1981) under resampling of the data. We have performed a delete-one jack-knife procedure (similar to leave-one-out CV) (miller, 1974), which a statistical technique closely related to bootstrap used to estimate sampling variability of statistics that would be otherwise hard or impossible to compute analytically. We repeated the whole feature selection and CCA procedure multiple times always with leaving one subject out of the analysis. This produces a distribution of canonical loadings and thus allows us to estimate their stability, and therefore uncertainty, under small perturbations of the data (here by exchanging one subject) taking into an account both the feature selection step and the CCA step. Perturbation of data by leaving one subject should be tiny compared to other resampling schemes, thus providing a very conservative estimate of the stability of the procedure. In other words, if the solution is not stable after removing only one subject, it will not be stable under more extensive resampling.

### 2.6 Clustering analysis

In the original study, the first two canonical variates of the RS-fMRI connectivity features were used as input for the clustering analysis. The underlying idea was to constrain the clustering analysis to a low dimensional representation of brain connectivity features that are clinically relevant. The authors used a hierarchical clustering procedure using the Euclidean distance measure and Ward's D linkage method, which minimize the total within-cluster variance. The original study identified a solution with four clusters as the best clustering solution partly because the CH index (variance ratio criterion or Calinski-Harabasz index) was maximized for the four cluster solution.

We followed the same procedure but in addition to the CH index we also computed the silhouette index, which compares average within cluster distances to average distances between points from different clusters. We also performed an additional procedure to test whether the observed CH and silhoutte indices are unlikely to occur under null hypothesis of no clusters. Although the CH index was in the original study maximized for four cluster solution, this, by itself, is not a statistical test or evidence of the existence of four clusters. Specifically, we don't know if the derived CH index was significantly higher than what would have been expected under the null hypothesis of data with no underlying clusters. Here we devise a procedure, similar to the one proposed in Liu et al. (2008),to test the statistical significance of the observed CH index. In this procedure, the null hypothesis is that the data came from a single 2-dimensional Gaussian distribution (i.e. distribution with no underlying clusters). Specifically, first, we estimated a covariance matrix between the two canonical variates used for the clustering analysis. Second, we repeatedly took random samples of the size of our dataset (187) from a bivariate Gaussian distribution defined by this covariance matrix. Third, we ran the same hierarchical clustering procedure as we performed on the real data on each random sample and calculated the best obtained CH and silhouette index, thus obtaining an empirical null distribution of these indices. The *p*-value was then defined as a proportion of the calculated indices in the null distribution smaller than what we observed in the real data.

To make our analysis comparable to the original study, we decided to perform a clustering analysis on two different sets of canonical variates. First, as in the original study, we performed clustering analysis on the first two RS-fMRI connectivity canonical variates, which were the two RS-fMRI components with the highest canonical correlations. Second, because the first two components in our analysis may not correspond to the first two components identified in the original study, we visually selected two canonical variates that showed the most similar clinical profiles to those identified in the original study.

#### 2.6.1 Stability of clustering

To reliably interpret the derived clusters, it is important to evaluate if the clustering assignment is stable under small perturbations to the data. In other words, if the same procedure was repeated using a similar dataset, would we identify similar clusters and would we assign the same subjects to the same clusters? This is a different question than if the clusters are statistically significant, because cluster assignment might be stable even if there are no real clusters in the data. On the other hand, it is possible to obtain clearly distinct clusters that are unreliable and cannot be reproduced in a different dataset. For this, we employed the same leave-one-out procedure as for the estimation of the stability of the canonical loadings. The whole feature selection, CCA, and hierarchical clustering procedure were repeated for each subject, always with one subject left out of the training process. This allowed us to estimate the stability of the canonical variates under slight perturbation of the data and subsequently the stability of the whole clustering procedure that is based on these canonical variates. As noted above, perturbation of removing one subject should be weak, and therefore this provides a very conservative test of stability in the sense that if the solution is not stable under a leave-one-out procedure it will not be stable under a greater perturbation to the data.

### 2.7 Code availability

The code used to perform data analysis can be found in supplementary materials and also at https://github.com/dinga92/niclin2019-biotypes.

## 3 Results

### 3.1 Sample characteristics

Sample characteristics are provided in Table 2.

**Table 2:** Sample characteristics for the NESDA (stratified by study site) and MOTAR samples.

	NESDA 1	NESDA 2	NESDA 3	MOTAR	Total
N	32	57	62	36	187
Age (SD)	36 (9.52)	37.26 (10.26)	35.87 (11.51)	36.69 (12.37)	36.48 (10.93)
N Female (%)	19 (59%)	39 (68%)	43 (69%)	23 (66%)	124 (66%)
N MDD (%) (no anx. disorder)	13 (40%)	18 (32%)	22 (35%)	10 (28%)	63 (34%)
N Anxiety disorder (%) (no MDD)	11 (34%)	13 (22%)	21 (34%)	2 (6%)	47 (25%)
N Comorbid (%)	8 (25%)	26 (45%)	19 (30%)	24 (67%)	77 (41%)
Current⁎ IDS (SD)	22.31 (9.66)	24.66 (11.45)	19.93 (12.42)	15.81 (10.56)	21.11 (11.96)
Current⁎ IDS categorical none/mild/moderate/severe/very severe	5/16/9/1/1	10/18/22/6/1	22/20/16/2/2	19/10/5/2/0	56/64/52/11/4
Current⁎ BAI (SD)	13.19 (8.45)	13.33 (9.73)	13.34 (9.35)	21.67 (11.95)	14.91 (10.34)
Current⁎ BAI categorical none/mild/moderate/severe	11/3/7/1	22/19/13/3	27/18/12/5	5/11/11/9	65/61/43/18
N in remission (IDS and BAI)	3 (9%)	10 (18%)	16 (25%)	5 (14%)	34 (18%)
Baseline antidepressant use	7 (21%)	20 (35%)	23 (37%)	0 (0%)	50 (26%)
Social phobia	12 (37%)	23 (40%)	23 (37%)	15 (42%)	73 (39%)
Panic with agoraphobia	6 (18%)	13 (22%)	17 (27%)	8 (22%)	44 (24%)
Panic without agoraphobia	4 (13%)	11 (19%)	9 (15%)	1 (3%)	25 (13%)
Agoraphobia	4 (13%)	3 (5%)	3 (5%)	0 (0%)	10 (5%)
GAD	7 (22%)	16 (28%)	15 (24%)	7 (19%)	45 (24%)

Anxiety disorder: one or more of panic disorder, generalized anxiety disorder, social phobia, and agoraphobia. IDS: inventory of depressive symptomatology. BAI: Beck anxiety inventory. GAD: generalized anxiety disorder. IDS categorical cutoffs: 0–13/14–25/26–38/39–48/49–84. BAI categorical cutoffs (Kabacoff et al., 1997): 0–9/10–18/19–29/30–63.

⁎ At the time of scanning.

### 3.2 CCA significance

**Fig. 2:**
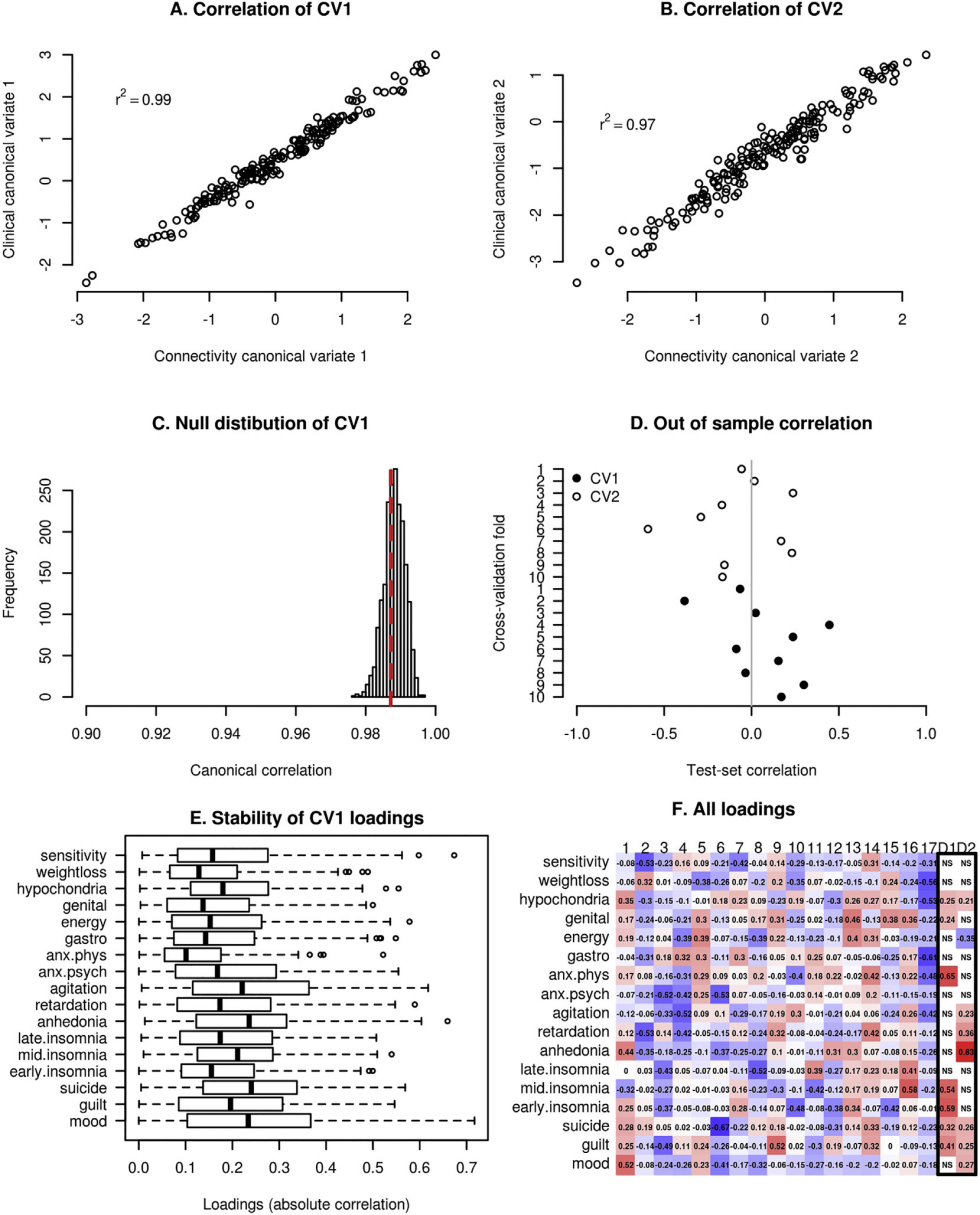
A, B) CCA finds a linear combination (canonical variate) of brain connectivity features that maximizes correlation with a linear combination of clinical symptoms. Canonical correlations are high and comparable to the original study (0.95 and 0.91). C) The null distribution of the first canonical correlation obtained using permutation test. Although canonical correlations in A and B are seemingly high, they are also high under the null hypothesis and thus not statistically significant. D) Out of sample canonical correlation for first two canonical pairs estimated by 10 fold cross-validation. Each point represents out of sample canonical correlation for each cross-validation fold. Although the canonical correlation was high in the training set as showed in A and B, id dropped to a chance level correlation in the test sets. E) Canonical loadings for the first canonical variate and their stability under resampling of the data using leave-one-out (jack-knife) procedure. F) Clinical canonical loadings for all canonical variates (1–17) and first two reported in the original study (D1-D2).

Canonical correlations were 0.99 and 0.97 for the first two pairs of canonical variates. As can be seen in the null distribution provided in Fig. 2, canonical correlations this high are not unusual even if there is no actual correspondence between X and Y (as determined by a permutation test). Indeed, the respective *p*-values of the permutation tests were not significant (*p* = 0.64 and *p* = 0.99), neither were they significant according to the Wilk's lambda statistics (p = 0.99 and p = 0.99), which measured the significance of the whole decomposition. Our permutation testing procedure took into account that connectivity features had been selected based on their correlation with clinical features. In contrast, *p*-values computed analytically using traditional chi-square approximation of the null distribution of the Wilk's lambda statistics with pq degrees of freedom, p and q being the number of variables in X and Y, were < 0.0001. Because the Drysdale et al. study did not test the significance of their CCA solution in the same way, it remains to be confirmed whether the canonical variates identified in their original study were significant, although authors did provide indirect evidence of this using an independent validation sample.

#### 3.2.1 Out of sample canonical correlation

Using 10-fold cross-validation, the average out of sample canonical correlation across folds was 0.07 and −0.07 for the first and second canonical pair, respectively (see Section 2.5.2), in contrast to the within-sample canonical correlation of 0.99 and 0.97. Canonical correlations for each test fold can be seen in Fig. 2D. This shows that even a high within-sample canonical correlation might not necessarily be reproducible in an independent test set. The original study used an independent evaluation set of 92 subjects. However, the authors did not perform a CCA analysis in the independent sample directly and thus did not provide canonical correlations for the independent validation dataset. Instead, they demonstrated that subjects assigned to clusters in the validation set according to their RS-fMRI connectivity features showed similar clinical profiles as the clusters identified in the training set.

#### 3.2.2 CCA similarity of loadings

A side-by-side comparison of canonical loadings (univariate correlation between each variable and the canonical variate) of all our resulting canonical variates and the first two canonical variates reported in Drysdale et al. are provided in Fig. 2f. We also conducted an analysis of stability of these loadings under small resampling of the data by repeating the feature selection and CCA procedure 187 times, each time without one subject left out of the analysis. The results for this analysis can be seen in Fig. 2e. It can be seen that even by changing one subject in the pipeline, individual loadings changed dramatically. Because of this instability and the fact that our canonical variates were not statistically significant, it was not found meaningful to compare our loadings with loadings found in the original study.

### 3.3 Clustering significance

**Fig. 3:**
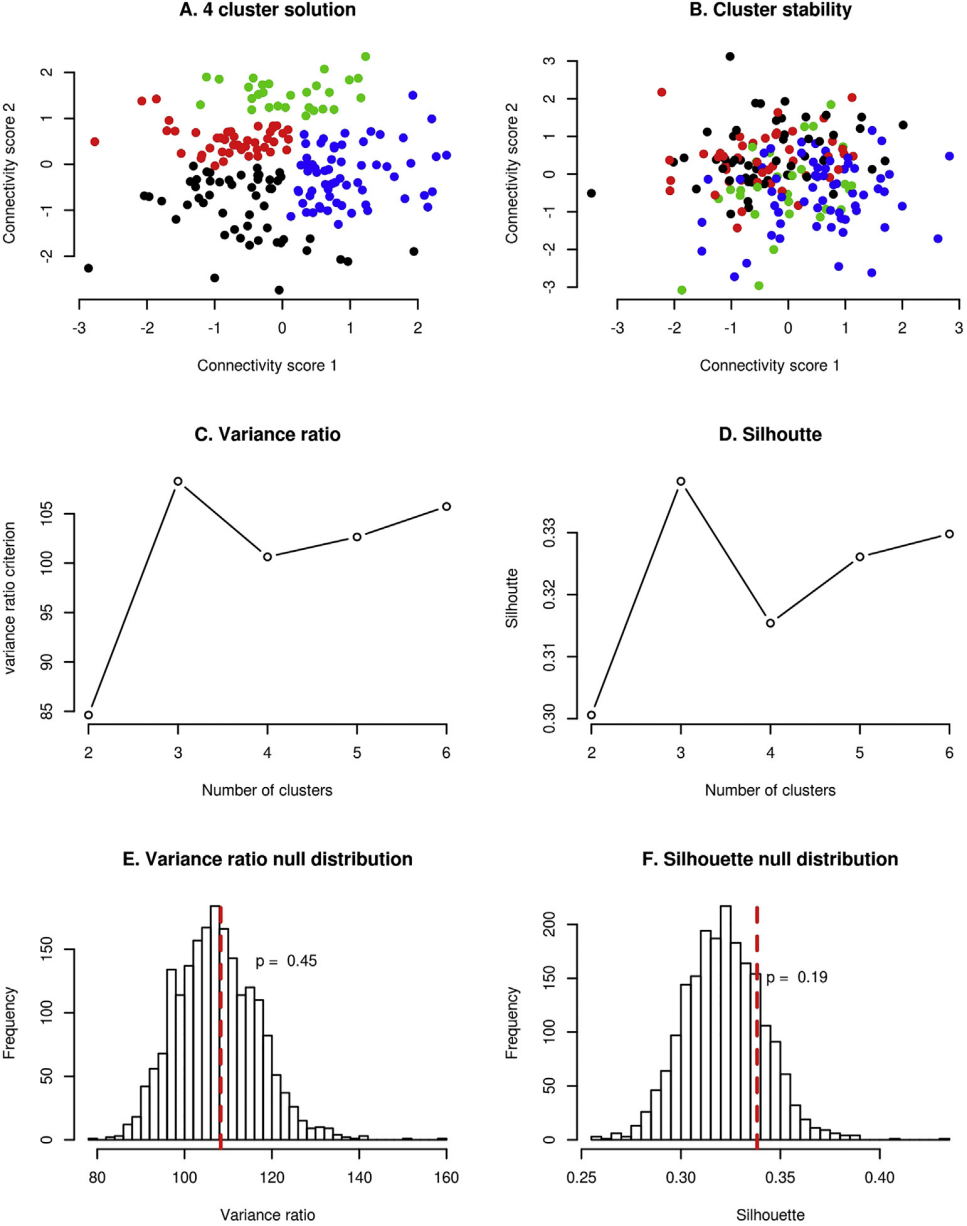
A) obtained 4-cluster solution using hierarchical clustering. B) Stability of the cluster assignment. Each subject is shown with the same color as it had in A, but the connectivity scores are recomputed under a small perturbation of the data i.e. leaving one subject out of the feature selection and CCA procedure. C) Variance ratio criterion is maximized at 3 clusters (4 in the original study). D) Silhouette index is maximized at 3 clusters. E, F) Null distribution of Variance ratio and silhouette indices. Showing that although these indices are maximized at 3 clusters, these results are not unusual even for the data simulated from a distribution with no clusters. Therefore these criteria do not imply evidence for the existence of clusters in our data or in the data presented in the original study.

In our dataset, a 3-cluster solution showed the highest CH (109) and also the highest silhouette index (0.34) (Fig. 3C and D, respectively). However, using a simulation approach described in the methods section, these indices were not statistically significant (*p* = 0.45 and *p* = 0.19 for CH and silhouette index, respectively). That means that it is not unusual to observe such high CH and silhouette indices, even when the hierarchical clustering is performed on a normally distributed data set (data with no clusters). Formally, this means that we cannot reject the null hypothesis of the data coming from a single Gaussian distribution (Fig. 3). In the original study, this was not tested. Therefore, we cannot say if the data in the original study really formed clusters, instead of just random fluctuation of the data.

#### 3.3.1 Cluster stability

We evaluated cluster assignment stability under slight resampling of the data by performing the feature selection and CCA procedure again, with one subject left out of the pipeline. The results are shown in the Fig. 3a and b. The figures show the position of each subject with respect to the first two canonical variates and therefore how clusters changed just by changing one subject in the pipeline. Stability of cluster assignment was performed in the original study by bootstrap procedure after feature selection and CCA, thus not taking instability of these two steps into account.

#### 3.3.2 Clustering similarity

Since we did not find evidence for clusters in our data, and the cluster assignment was not stable, we did not consider it meaningful to describe clusters in terms of their symptom profiles or compare the clusters in our study to clusters identified in the original study.

## 4 Discussion

Here, we aimed to replicate the analytical pipeline proposed by Drysdale and colleagues (Drysdale et al., 2017) and extend their findings to a different clinical population. More specifically, we applied their stratification approach to a similarly sized dataset of a heterogeneous sample of patients with depression and anxiety disorders, i.e. with different clinical characteristics to the original sample (which included only subjects with currently active treatment-resistant depression) and with clinical symptoms measured with a different but comparable instrument. We followed the analysis steps of the original study as closely as possible and obtained extremely high canonical correlations (0.99 and 0.97) and optimal 3 cluster solution. However, after performing additional tests that were not performed in the original study, which took into account that the RS-fMRI connectivity features were selected based on their correlation with clinical features before performing CCA on these connectivity and clinical features, even the high canonical correlations that we observed were not statistically significant and they did not replicate outside of the training set. By using the same criteria for selecting the number of clusters as in the original study, we found an optimal three cluster solution. However, we showed that this cluster solution would happen even if the data came only from a single Gaussian distribution with no underlying clusters.

### 4.1 Statistical significance of canonical correlations

The first two canonical correlations between brain connectivity and clinical symptom measures that we observed in our data were high (0.99 and 0.98). However, they were not statistically significant as determined by permutation testing. In addition, using cross-validation, the canonical correlations dropped to approximately 0 in the test set. This is not unexpected because of the high number of variables included compared to the number of subjects in this sample, which leads to severe overfitting. CCA is known to be unstable; for example, introductory texts recommend between 10 and 42 subjects per variable in order to obtain a reliable CCA model (Tabachnick and Fidell, 2001; Pituch et al., 2016), but the pipeline used in the original study that we followed had around 1.3 subjects per variable. Another important contribution for the overly optimistic canonical correlations is the initial feature selection step that selected 150 connectivity features in our study (178 in the original study) out of ~30,000 brain connectivity measures that were most correlated with the clinical symptoms in the same dataset in which the CCA was performed.

In the original study by Drysdale and colleagues, this feature selection step was not taken into account when estimating the statistical significance of the canonical correlations, thus the reported *p*-values were likely inflated. Moreover, the replication of canonical correlations out of sample was not shown directly in the study by Drysdale and colleagues. Despite this, the authors did provide indirect evidence for a reliable relationship between brain connectivity measures and clinical symptoms in a subset of subjects left out completely from the primary analysis (training set). These subjects were assigned to clusters based on a support vector machine classifier using only their connectivity features as predictors, and these clusters had similar clinical symptom profiles in their cluster discovery and replication sets, which would not be possible if there is no real relationship between biological and clinical variables. However, in view of the methodological considerations above, this does necessarily mean that the relationship is exactly in the form of anxiety and anhedonia-related components as reported in the original study.

### 4.2 Similarity of canonical loadings

Due to the overfitting of CCA discussed above, the canonical loadings we obtained were unstable, which makes their comparison with loadings reported in the original study difficult. Despite that, loadings of our fourth canonical variate were most similar to the loadings of the second canonical variate reported in the original study by Drysdale and colleagues. However, our canonical variates were not statistically significant.

### 4.3 Clustering analysis

A problem with many clustering algorithms that is not commonly recognized is that they always yield clusters, regardless of the structure of the data, even if there are no clusters at all (Liu et al., 2008). Many procedures employed to determine the optimal number of clusters, including the one used in the original study, are therefore more heuristic and do not provide a statistical test of the underlying structure of the data. In the original study, a four-cluster solution was decided to be optimal mainly because the CH criterion, a specific numerical value describing how well the data form clusters, was maximized by four clusters. According to this criterion, we would have chosen an optimal number of clusters to be three (or two according to the silhouette criterion) in our data. However, after a closer examination, we observed that a CH index as high or higher as we observed, can be easily obtained just by running the same hierarchical clustering procedure on data randomly sampled from a distribution that does not contain any clusters (in this case Gaussian distribution). Or in other words, according to the CH index, we could not reject the null hypothesis that our data came from a single Gaussian distribution. No test for the existence of clusters was performed in the original study and the presented data in Fig. 1 of the original study looked more like a continuous distribution instead of 4 clusters. On the basis of these methodological considerations, we think that the original study provided insufficient evidence to conclude that any number of distinct biotypes of depression was more likely than no biotypes at all.

Although the absence of clusters would change the conclusion of the original study, it is not necessarily detrimental to the significance of the results. The found biological axes related to different depressive symptoms are important in their own merit, without subsequent arbitrary dichotomization into four biotypes. Two canonical variates already provide a parsimonious representation of the data and dividing them further into four subtypes would not provide any more insights into mechanisms of depression (especially if these subtypes are spurious).

Assuming biotypes is also detrimental for the sake of clinical utility, such as predicting the probability of a TMS treatment response. Since the subtypes were predictive of TMS treatment response and were based on the underlying canonical variates, it is reasonable to assume that the probability of response varies smoothly with respect to the canonical variates. Using only discrete subtypes for prediction assumes that all the subjects in one “biotype” have the same chance for response. Also, very similar subjects might get significantly different predictions. If a subject would move slightly from biotype 1 to bordering biotype 2, his predicted TMS response chance would jump from 80% to 20%. Even if the biotypes were truly distinct, but the probability of a response would vary smoothly with respect to canonical variates, instead of discretely with respect to subtypes, the model using continuous variables as predictors will perform better than a model using only subtype information.

On the other hand, using subject-specific connectivity scores alone, without additional arbitrary dichotomization into biotypes, would allow making an individualized prediction for each individual, in line with goals of personalized precision medicine. A clinical decision can then be made for each patient individually according to their treatment response probability instead of the average treatment response probability of the whole group (e.g. biotype). Critically, the availability of quantitative measures means that cut-off points for various levels of severity can be changed and fine-tuned as more data from future studies become available — as has been done for diseases such as hypertension. Severity cut-points explicitly acknowledge dimensions and move away from traditional single disorder models. Such a dimensional approach, which captures the full spectrum of brain connectivity alterations, provides an empirical and coherent framework to accommodate comorbidity and sub-threshold symptoms.

Due to the clinical heterogeneity of many psychiatric disorders and the quest for personalized medicine, there is a tendency towards subtyping and expanding psychiatric nosology. However, the presumption of distinct and homogeneous subtypes might not be clinically useful and might not represent the underlying biology. Many clustering approaches will always produce some clusters and would so even for uniformly distributed data. It is, therefore, crucial to distinguish real biologically or clinically meaningful subtypes from random fluctuation of the data. This is not an easy task, however, several methods exist. One possibility is to use simulations to create an empirical null distribution of clustering statistics, similar to what we used here as proposed by (Liu et al., 2008) or use model-based approaches, such as latent class analysis or Gaussian mixture models, where the model fit can be tested directly.

### 4.4 Recommendations for future studies

We have several recommendations for future studies. First, to avoid overfitting and unstable results of CCA, we advise to either reduce the number of features to limit the feature to sample ratio which can be done by using a dimensionality reduction method, such as PCA, ICA or factor analysis as used in Smith et al. (2015), or to use a regularized version of CCA, or both as used in Le Floch et al. (2012). Second, if a feature selection step is involved, it is necessary to take this into account in the statistical testing procedure, either by doing a statistical test in an independent test set or by incorporating this selection step into a permutation testing procedure (Kriegeskorte et al., 2009). For clustering analysis, it is necessary first to answer the question if there are actually real clusters in the data or just random fluctuations. Clustering coefficients and cluster assignment stability evaluation do not test for this. To estimate cluster stability assignment it is important to take the whole procedure into account, including feature selection and CCA, which might show that even seemingly stable clusters are unstable. Finally, if the goal is a clinically useful prediction and high accuracy, continuous variables should be preferred before dichotomizing data into clusters because they contain more information and thus lead to better prediction.

### 4.5 Limitations

There are several potential limitations mostly arising from our study not exactly matching the original study, thus this study cannot be considered a direct replication. Non-exact replications like the current study provide a high level of evidence for the robustness and external validity of the original finding if they are replicated because they show that the original results do not depend on specific study design choices and that they can be generalized to different populations(Rosenthal, 1990) On the other hand, if they fail to support the original findings, it is hard to differentiate whether this failure is due to differences between studies or because the original finding was a false positive. Our study differed from the original in some preprocessing steps, the clinical instrument used to assess depressive symptoms and clinical characteristics of the sample.Our preprocessing pipeline differed in several ways, the most notable was our choice to handle motion-related artifacts using ICA-AROMA instead of motion scrubbing. It has been shown repeatedly that AROMA performs at least as well as motion scrubbing in reducing motion-related artifacts (Pruim et al., 2014; Parkes et al., 2018), with the additional benefit of keeping the number of available timepoints constant across subjects. It is possible that different preprocessing choices would yield different results, however, biological effects should not disappear by changing to an analogous preprocessing pipeline. An additional difference is that our data were obtained from four scan-sites with different average symptom severity, compared to two scan -sites with similar average symptom severity in the original study. This limits the statistical power of our analysis because correcting for site effects will remove all depression-related connectivity effects in the data that can be explained by site effects. Especially subjects from the MOTAR study have on average one standard deviation lower depression severity symptoms than the rest of the subjects from the remaining 3 study sites. However, this does not affect our main conclusions about the tendency of the procedure used in the original study to produce overly optimistic results.In the present study, the assessment of clinical symptoms was performed using the self-report IDS instrument compared to a clinician-administered (HAM-D) used the original study. Both instruments are widely used and represent gold-standard rating scales for depression in clinical research (Cusin et al., 2009). Responses to the two different instruments are highly correlated, and their specific items are related to mostly overlapping symptoms (Rush et al., 1996; Gullion and Rush, 1998). In general, replication of effects using different instruments provides evidence that the found association is characteristic of the pathology rather than the specific instrument. Specifically, the original study found an association with biology and anxiety, which should not depend on the precise way in which specific anxiety-related items were worded. That being said, different items, although measuring mostly the same symptoms, are likely to have a different correlation structure, which in turn might produce different loadings in CCA. Indeed we did not find the exact same loadings as in the original study, however, we have also shown that the CCA procedure was unstable due to overfitting. Therefore, a direct comparison between loadings observed in our study and the original study is was found not meaningful regardless of the instrument being used.Our sample characteristics were different from the original study, which included only subjects with a currently active episode of a treatment-resistant major depressive disorder. In contrast, we included subjects with a wider range of diagnoses, including MDD and anxiety disorders and a wider range of symptom severity from sub-threshold depression (i.e. MDD subjects in remission at the time of scanning) to people with an active MDD episode with mild, moderate and severe symptoms. Our sample was recruited from the general population, primary care and specialized mental health care. We believe that the inclusion of pure anxiety disorder patients is especially appropriate because the original study identified an anxiety associated CCA component and an anxiety-associated biotype. Moreover, the authors of the Drysdale study also applied their model to patients with generalized anxiety disorder and concluded that connectivity abnormalities overlapped significantly with those in patients with depression. For the generalizability of the original findings, it is important to show that any found association exists also in a population in a naturalistic setting where the depressive phenotype is simultaneously less severe, more heterogeneous and more reflective of the variability in the MDD phenotype in the general population. Studies showed a difference in resting state activity between remitted and actively depressed subjects (Cheng et al., 2019; Dong et al., n.d.). A wider range of symptoms could, on one hand, translate to more diverse biology and thus make the connection between clinical symptoms to biology more apparent. On the other hand, higher heterogeneity might have the opposite effect if the underlying biological mechanisms differ. Specifically, the power of our study to find significant effects would be decreased if specific symptoms have different effects on functional connectivity in different groups of patients (i.e., active and non-active depressive disorder or depressive disorder and anxiety disorder). However, our main conclusions of potential unreliability of the original findings are not based on observing a different effect as in Drysdale et al., but are based on performing additional sufficiently rigorous statistical tests, thus showing that the analytical methods used in the original study can produce extremely high and seemingly statistically significant canonical correlations even when there is no relationship between data modalities and a clustering procedure that will always produce some clusters regardless of the input data.

## 5 Conclusion

To the best of our knowledge, this is the first attempt to replicate the methodological pipeline used in Drysdale et al. and to extend it to a different clinical sample of patients with depression and anxiety disorders. Extending the original methods with additional more rigorous statistical procedures, we did not find stable or statistically significant biotypes of depression and anxiety in an independent sample from a different clinical population. Furthermore, we have argued that the evidence for the existence of 4 distinct biotypes presented in the original study, namely stability of the clustering solution and magnitude of the clustering index, is not sufficient to conclude (or disprove) the existence of distinct biotypes and the results should therefore, be interpreted with care. However, even without partitioning patients into biotypes, the existence of continuous biological axes related to symptoms of depression might be even more useful for our understanding of biological mechanisms of depression and clinical practice. This conclusion is not based on the direct comparison of results between the present study and Drysdale et al. (which would not be possible due to sample differences), but it is based on our evaluation of statistical properties of the methods used in the original study.

## Appendix A Supplementary data

Supplementary materials: supplementary figures and supplementary analysis. Image 1

Analysis code. Image 1
