# Supplementary material for: Evaluating the evidence for biotypes of depression: Methodological replication and extension of Drysdale et al. (2017)
Source: Neuroimage Clin. 2019 Mar 27;22:101796. doi: 10.1016/j.nicl.2019.101796 (PMC6543446; doi:10.1016/j.nicl.2019.101796)
Supplement: Supplementary file 1 — Supplementary materials: supplementary figures and supplementary analysis. [file mmc1.doc]

**Supplementary materials**

**
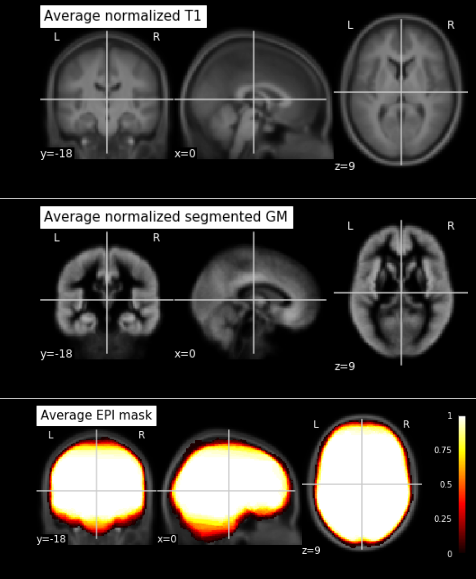
**

**Supplementary figure 1:** Quality of normalization and average brain coverage. Average normalized T1: is a mean image of all T1 scans normalized to standard MNI space. Average normalized segmented GM: Is a mean image of all subject specific gray matter segmentation. Average EPI mask: an average of all extracted brain masks normalized to MNI space demonstrating a degree of brain coverage in our data.

**
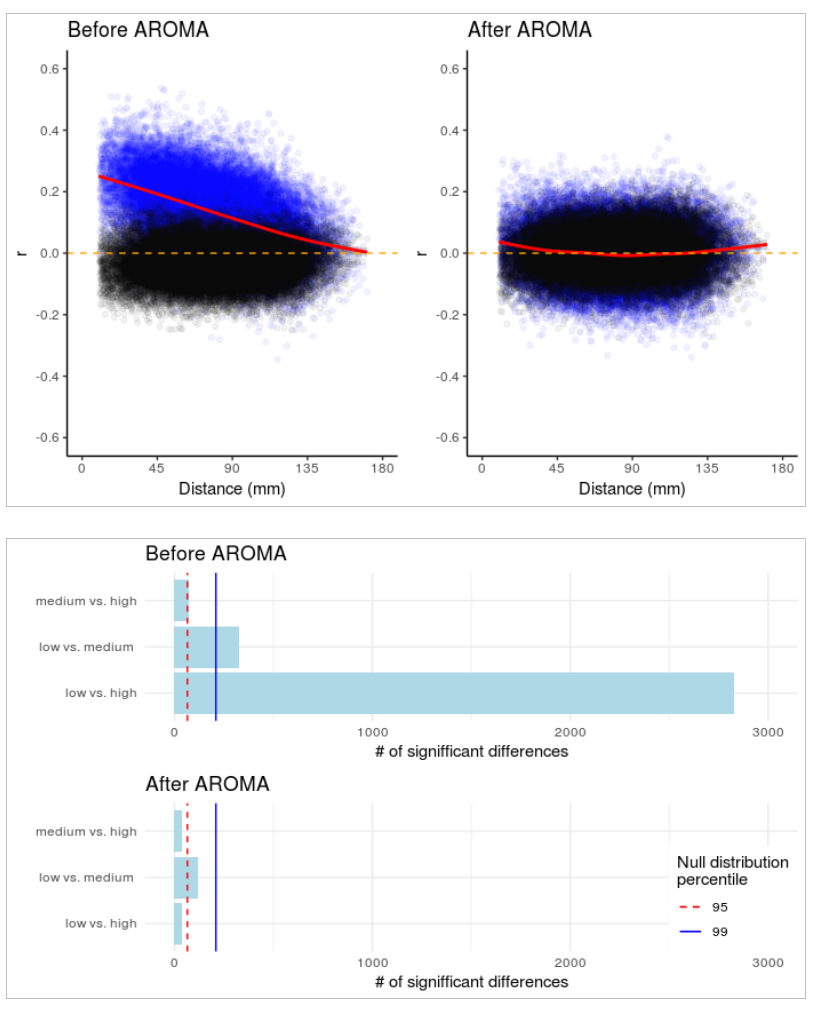
**

**Supplementary figure 2:** Correlation of anatomical distance between brain nodes and a correlation between their connectivity and motion measured as an average frame-wise displacement. This is indicative of the severity of motion-related artifacts and the success of our cleaning strategy (using ICA AROMA). Bellow, the pair-wise comparison between low moving, medium moving, and high moving subjects dichotomized using tertile split. For each voxel, statistical significance was obtained using a t-test, then permutations were performed to compute null distribution of voxels with statistically significant (p < 0.005) differences. Lines show 95th and 99th percentiles of this null distribution, thus corresponding to p = 0.05 and p = 0.01.

**
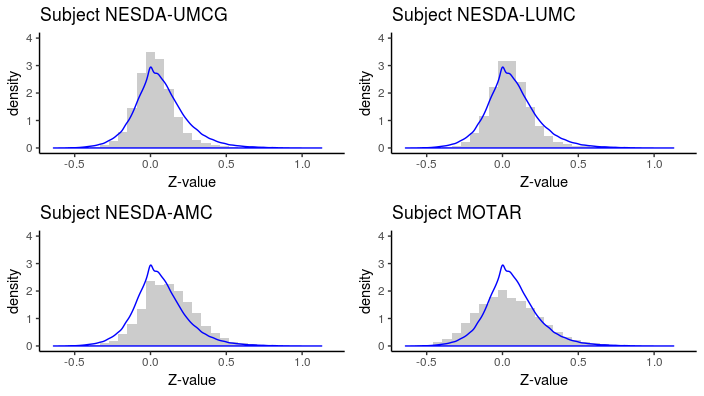
**

**Supplementary figure 3:** Distribution of Fisher’s z transformed between region connectivities of all subjects (blue) and of one selected subject per scan-site (gray). Distributions were centered approximately at 0 and were similar between samples.


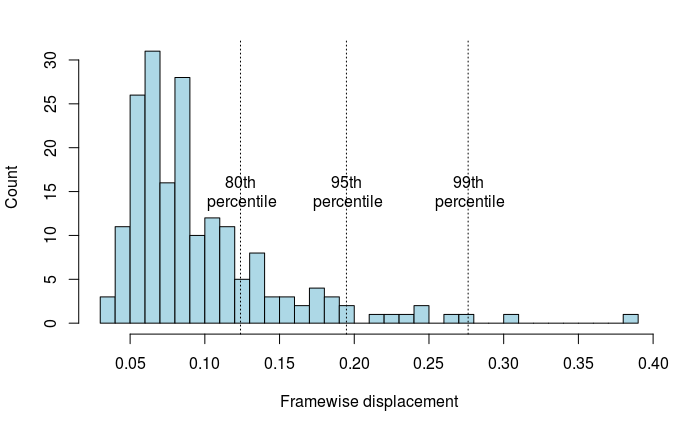


**Supplementary figure 4:** Distribution of head motion measured by an average displacement of each volume at a specific time-point from the reference image. 80% of subjects had an average framewise displacement bellow 0.12 mm.

**Supplementary analysis**

We have repeated all analysis steps reported in the manuscript in a subsample of patients (N=140) more representative of the sample in the original study i.e we have included only subjects with MDD or MDD-anxiety disorder co-morbidity, excluding pure anxiety disorder patients. We changed the number of selected variables from 150 to 112 in order to keep the same ratio of selected features to a number of subjects.

First, two canonical correlations were 0.997 and 0.995 respectively. This was not significant using according to a permutation test of canonical correlations (p=0.1 and 0.01 for first and second canonical correlation respectively) or Wilk’s statistic (p=0.17 p=0.24). The average of canonical correlations in the cross-validation was 0.02 and -0.04 for the first two canonical correlations. The optimal number of clusters was 3 according to CH index and also silhouette index, but the clusters were not statistically significant (p=0.43 and p=0.2 for CH and silhouette index respectively).

This shows that even in the more homogeneous sample, CCA overfits the data, and it is not reproducible outside of a training set.
