# Supplementary material for: Evaluating the evidence for biotypes of depression: Methodological replication and extension of Drysdale et al. (2017)
Source: Neuroimage Clin. 2019 Mar 27;22:101796. doi: 10.1016/j.nicl.2019.101796 (PMC6543446; doi:10.1016/j.nicl.2019.101796)
Supplement: Supplementary file 2 — Analysis code. [file mmc2.pdf]

Supplementary material - data analysis code

Contents

0. Data loading and preparation **1**
Load data . . . 1
Summary statistics . . . 2
Delete variables with too many missing values . . . 3
Fisher Z transform RS connectivity measures . . . 3
Regress out age and scan location and framewise displacement . . . 3
Median impute missing data . . . 3

1. Canonical correlation analysis **4**
Feature selection and CCA function . . . 4
Canonical correlations . . . 4
Permutation test . . . 6
Cross-validation . . . 8
Stability of canonical loadings . . . 10
Compare loadings with original study . . . 12

2. Clustering analysis **13**
Run hierarchical clustering . . . 13
Stability of cluster assignment . . . 13
Clustering indeces . . . 14
Statisticall significance of clusters . . . 15

3. Software enviroment **17**

0. Data loading and preparation

Load data

```
library(data.table)
rs_connectivity <- as.data.frame(fread('./rs_connectivity_prepared.csv'))
clinical <- read.csv('./clinical_prepared.csv')
nuisance_covs <- read.csv('./nuisance_covs_prepared.csv')
```

Print number of rows of each loaded dataframe

```
c('rs_connectivity' = nrow(rs_connectivity),
  'clinical' = nrow(clinical),
  'nuisance_covs' = nrow(nuisance_covs))
```

```
## rs_connectivity        clinical   nuisance_covs
##             187             187             187
```

Are subjects in data frames in the same order?

```
all(rs_connectivity$pident == nuisance_covs$pident)
```

```
## [1] TRUE
```

```r
all(rs_connectivity$pident == clinical$pident)
```

```
## [1] TRUE
```

Throw away the subj.id column, because it is not needed anymore

```r
rs_connectivity <- rs_connectivity[, names(rs_connectivity) != 'subj.id']
clinical <- clinical[, names(clinical) != 'subj.id']
nuisance_covs <- nuisance_covs[, names(nuisance_covs) != 'subj.id']
```

Recode factor variables

```r
nuisance_covs$sex <- as.factor(nuisance_covs$sex)
nuisance_covs$scan.location <- as.factor(nuisance_covs$scan.location)
```

Summary statistics

Summary of all subjects

```r
summary(nuisance_covs)
```

```
##       age         sex     scan.location    diagnosis   frame.displacement
##  Min.   :18.00   1: 63   MOTAR  :36    anxiety :47   Min.   :0.03501
##  1st Qu.:27.00   2:124   NESDA.1:32    comorbid:77   1st Qu.:0.12585
##  Median :36.00           NESDA.2:57    mdd     :63   Median :0.18647
##  Mean   :36.48           NESDA.3:62                  Mean   :0.23318
##  3rd Qu.:45.00                                       3rd Qu.:0.28910
##  Max.   :67.00                                       Max.   :0.93380
```

Summary of MOTAR subjects

```r
summary(nuisance_covs[nuisance_covs$scan.location == "MOTAR",])
```

```
##       age         sex    scan.location    diagnosis   frame.displacement
##  Min.   :22.00   1:13   MOTAR  :36    anxiety : 2   Min.   :0.07686
##  1st Qu.:27.00   2:23   NESDA.1: 0    comorbid:24   1st Qu.:0.18018
##  Median :34.00          NESDA.2: 0    mdd     :10   Median :0.24107
##  Mean   :36.69          NESDA.3: 0                  Mean   :0.30244
##  3rd Qu.:46.25                                      3rd Qu.:0.34256
##  Max.   :67.00                                      Max.   :0.83957
```

Summary of NESDA subjects

```r
summary(nuisance_covs[nuisance_covs$scan.location != "MOTAR",])
```

```
##       age         sex     scan.location    diagnosis   frame.displacement
##  Min.   :18.00   1: 50   MOTAR  : 0    anxiety :45   Min.   :0.03501
##  1st Qu.:27.50   2:101   NESDA.1:32    comorbid:53   1st Qu.:0.12129
##  Median :37.00           NESDA.2:57    mdd     :53   Median :0.17712
##  Mean   :36.42           NESDA.3:62                  Mean   :0.21666
##  3rd Qu.:45.00                                       3rd Qu.:0.25216
##  Max.   :57.00                                       Max.   :0.93380
```

SD age: All/MOTAR/NESDA

```r
sd(nuisance_covs$age)
```

```
## [1] 10.92857
```

```r
sd(nuisance_covs[nuisance_covs$scan.location == "MOTAR",]$age)
```

```
## [1] 12.37005
```

```r
sd(nuisance_covs[nuisance_covs$scan.location != "MOTAR",]$age)
```

```
## [1] 10.6009
```

Delete variables with too many missing values

```r
rs_connectivity[rs_connectivity == 0] <- NA
# is.na(rs_connectivity) <- !rs_connectivity

num_na <- colSums(is.na(rs_connectivity))
rs_connectivity <- rs_connectivity[,num_na < 20]
```

Fisher Z transform RS connectivity measures

```r
library(psych)
ztransformed_rs_connectivity <- fisherz(rs_connectivity)
```

Regress out age and scan location and framewise displacement

```r
residual_rs_connectivity <- matrix(NA,
                                   nrow = nrow(ztransformed_rs_connectivity),
                                   ncol = ncol(ztransformed_rs_connectivity))

for (i in 1:ncol(ztransformed_rs_connectivity)) {
  fit <- lm(ztransformed_rs_connectivity[,i] ~ age + factor(scan.location) +
              frame.displacement,
            data = nuisance_covs, na.action = na.exclude)
  residual_rs_connectivity[,i] <- residuals(fit)
}

residual_rs_connectivity <- data.frame(residual_rs_connectivity)
names(residual_rs_connectivity) <- names(residual_rs_connectivity)
rm(ztransformed_rs_connectivity)
rm(rs_connectivity)
```

Median impute missing data

```r
library(caret)
imputation <- preProcess(clinical, method = 'medianImpute')
clinical <- predict(imputation, clinical)
imputation <- preProcess(residual_rs_connectivity, method = 'medianImpute')
residual_rs_connectivity <- predict(imputation, residual_rs_connectivity)
```

1. Canonical correlation analysis

Feature selection and CCA function

Here we create a function that first selects resting state features (X) with the highest spearman correlation with any of clinical symptoms (Y) and then fits and returns a CCA model. This function will be used to compute canonical correalotions and also later for permutation test and cross-validation.

```
select_and_cca_fit <- function(X, Y, n_selected_vars){
  library(candisc)
  #select
  correlations <- cor(Y, X, method = "spearman")
  correlations <- apply(correlations, 2, function(x){max(abs(x))})
  corr.threshold <- sort(correlations, decreasing = T)[n_selected_vars]
  selected.X <- correlations >= corr.threshold
  selected.X <- X[,selected.X]
  #cca fit
  cca_model <- candisc::cancor(selected.X, Y)
  #return fitted model containing canonical correlations and wilks lambdas
  return(cca_model)
}
```

Canonical correlations

Fit the feature selection and CCA model, selecting 150 features and print all canonical correlations

```
n_selected_vars <- 150
cca_model <- select_and_cca_fit(residual_rs_connectivity,
                                clinical,
                                n_selected_vars)
```

```
## Loading required package: car

## Loading required package: carData

##
## Attaching package: 'car'

## The following object is masked from 'package:psych':
##
##     logit

## Loading required package: heplots

##
## Attaching package: 'candisc'

## The following object is masked from 'package:stats':
##
##     cancor
```

```
cca_model$cancor
```

```
##  [1] 0.9871545 0.9739202 0.9677686 0.9663348 0.9611734 0.9441874 0.9373012
##  [8] 0.9271186 0.9133999 0.9091458 0.8882932 0.8787111 0.8627911 0.8273523
## [15] 0.8030797 0.7579703 0.6713721
```

Create a function to compute canonical variates

```
predict.cancor <- function(cancor.obj, X, Y){
  X_pred <- as.matrix(X) %*% cancor.obj$coef$X
  Y_pred <- as.matrix(Y) %*% cancor.obj$coef$Y
  XY_pred <- list(X_pred, Y_pred)
  names(XY_pred) <- c("X_pred", "Y_pred")
  return(XY_pred)
}
```

Visualize canonical correlations

```
canonical.variates <- predict.cancor(cca_model,
                               residual_rs_connectivity[,cca_model$names$X],
                               clinical)
cca_y_loadings <- cor(clinical, canonical.variates$Y_pred)

par(mfrow=c(1,2))
plot(canonical.variates$X_pred[,1],
     canonical.variates$Y_pred[,1],
     bty='n',
     xlab='Connectivity canonical variate 1',
     ylab='Clinical canonical variate 1')
text(-2, 1,  bquote(r^2 == .(round(cca_model$cancor[1], 2))))

plot(canonical.variates$X_pred[,2],
     canonical.variates$Y_pred[,2],
     bty='n',
     xlab='Connectivity canonical variate 2',
     ylab='Clinical canonical variate 2')
text(-2, 2,  bquote(r^2 == .(round(cca_model$cancor[2], 2))))
```

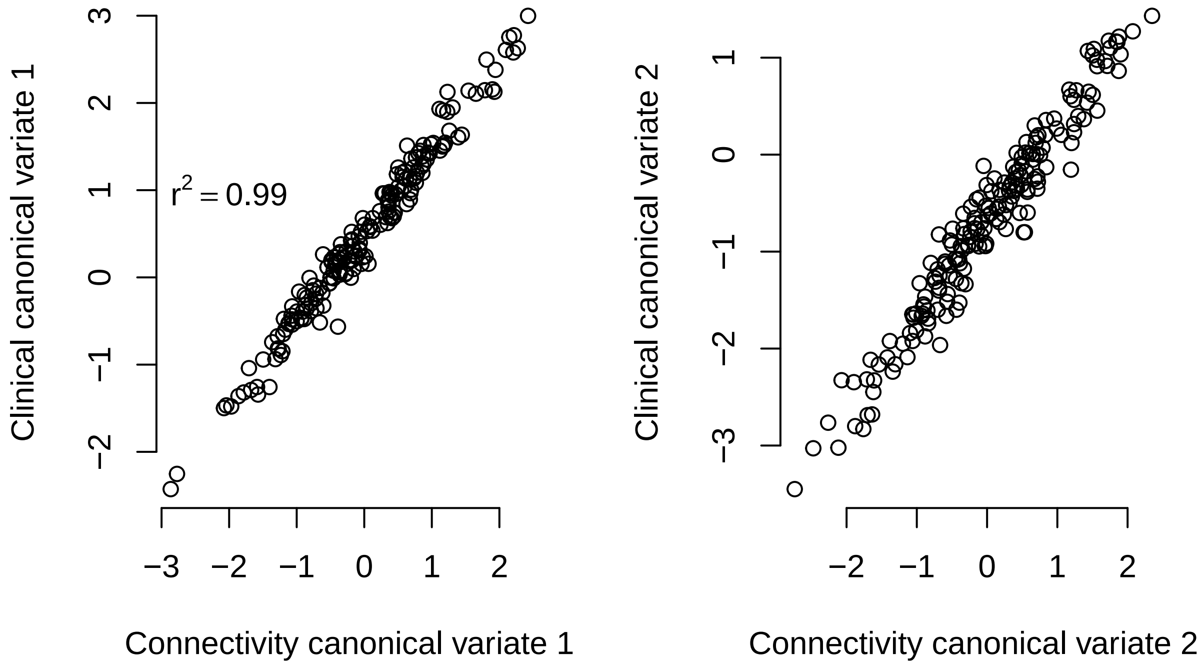

Permutation test

First get test statistics (canonical correlations and Wilks lambdas) from the real model

```
real_model <- cca_model
real_results_cancor <- real_model$cancor
real_results_wilks <- Wilks(real_model)$"LR test stat"
```

Obtain null distribution of test statistics by permuting rows of clinical data

```
library(permute)
library(doMC)
```

```
## Loading required package: foreach

## Loading required package: iterators

## Loading required package: parallel
```

```
registerDoMC(cores=4) # to run it multicore

nperms = 1999
set.seed(123)
# shuffle within scan location
shuffled_indexes <- sapply(1:nperms, function(x){
                          shuffle(1:nrow(residual_rs_connectivity),
                          control = how(blocks=nuisance_covs$scan.location))})
```

```r
null_results <- foreach(i=1:nperms) %dopar% {
  null_model <- select_and_cca_fit(residual_rs_connectivity,
                                   clinical[shuffled_indexes[,i],],
                                   n_selected_vars)
  #return canonical correlations and wilks lambdas
  list(null_model$cancor, Wilks(null_model)$"LR test stat")
}

# transform null results lists to data frame
null_dist_cancor <- lapply(null_results, function(x){return(x[[1]])})
null_dist_wilks <- lapply(null_results, function(x){return(x[[2]])})
null_dist_cancor <- as.data.frame(do.call(rbind, null_dist_cancor))
null_dist_wilks <- as.data.frame(do.call(rbind, null_dist_wilks))

get_pval <- function(real, null_dist, better="smaller"){
  if (better == "smaller"){
    rank <- sum(real < null_dist) + 1
  }
  if (better == "bigger"){
    rank <- sum(real > null_dist) + 1
  }
  pval <- rank / (length(null_dist) + 1)
  return(pval)
}

pvals_cancor <- mapply(function(real, null_dist){
                         get_pval(real, null_dist, better="smaller")},
                       real_results_cancor,
                       null_dist_cancor)
pvals_wilks <- mapply(function(real, null_dist){
                        get_pval(real, null_dist, better="bigger")},
                       real_results_wilks,
                       null_dist_wilks)
```

Print p-values

```r
print(cbind("component"=1:length(pvals_cancor), pvals_cancor, pvals_wilks))
```

```
##       component pvals_cancor pvals_wilks
##  [1,]         1       0.6475      0.9980
##  [2,]         2       0.9900      0.9980
##  [3,]         3       0.9815      0.9955
##  [4,]         4       0.7880      0.9900
##  [5,]         5       0.6270      0.9930
##  [6,]         6       0.9790      0.9985
##  [7,]         7       0.9315      0.9945
##  [8,]         8       0.9350      0.9950
##  [9,]         9       0.9675      0.9940
## [10,]        10       0.7735      0.9915
## [11,]        11       0.9410      0.9945
## [12,]        12       0.8330      0.9945
## [13,]        13       0.8120      0.9980
## [14,]        14       0.9795      0.9990
## [15,]        15       0.9675      0.9980
```

```
## [16,]        16       0.9800      0.9990
## [17,]        17       0.9930      0.9930
```

Visualize null distributions and p-values for first two canonical correlations

```
par(mfrow=c(2,1))
for (i in 1:2){
  hist(null_dist_cancor[,i], breaks = 25, main = paste("Null dist corr CV", i),
       xlim=c(0.9,1))
  abline(v=real_results_cancor[i], col="red")
}
```

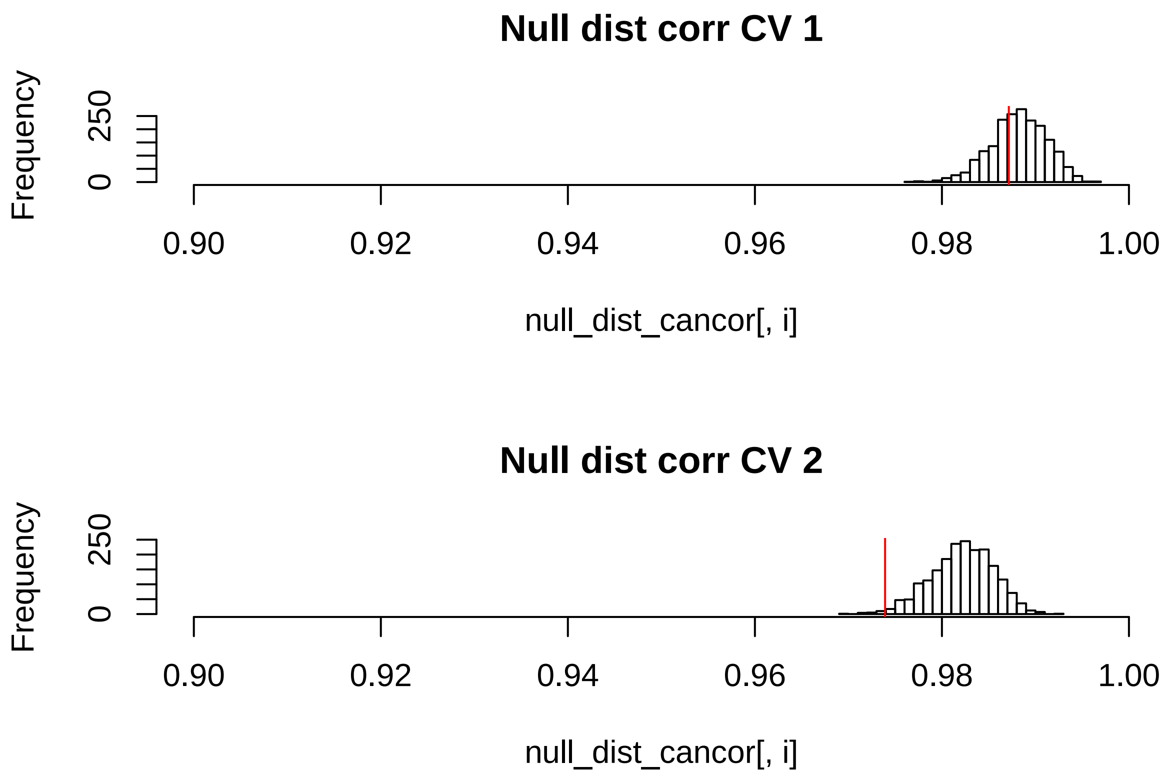

Cross-validation

Create function that performs cross-validation

```
cca_cv <- function(rs_variables, clinical_variables, n_selected_vars, site){
  library(caret)
  n_folds <- 10
  folds <- createFolds(as.factor(site), n_folds, list=F)
  results_cancor <- list()
  for (fold in 1:n_folds) {
    # create training and test set
    train_brain <- rs_variables[folds != fold,]
    train_clinical <- clinical_variables[folds != fold,]
    test_brain <- rs_variables[folds == fold,]
    test_clinical <- clinical_variables[folds == fold,]
```

```
    # fit on training set
    cancor.fit <- select_and_cca_fit(train_brain,
                                     train_clinical,
                                     n_selected_vars)
    # predict on test set
    XY_pred_cancor <- predict.cancor(cancor.fit,
                                     test_brain[,cancor.fit$names$X],
                                     test_clinical)
    results_cancor[[fold]] <- diag(cor(XY_pred_cancor[[1]],
                                       XY_pred_cancor[[2]]))
  }
  return(do.call(rbind, results_cancor))
}
```

Run cross-validation and print out of sample canonical correlations per CV fold for first two canonical variates

```
set.seed(123)
# we have 90% of subjects in the training set, so we will use 90% of variables
n_cv_selected_vars <- as.integer(n_selected_vars*0.9)
results_cca_cv <- cca_cv(residual_rs_connectivity,
                         clinical,
                         n_cv_selected_vars,
                         nuisance_covs$scan.location)
results_cca_cv <- results_cca_cv[,1:2]
colnames(results_cca_cv) <- c("CV1", "CV2")
results_cca_cv
```

```
##                CV1         CV2
##  [1,]  0.17096542 -0.16633571
##  [2,]  0.29947682 -0.15532348
##  [3,] -0.03464058  0.23144164
##  [4,]  0.15407033  0.16918998
##  [5,] -0.08714670 -0.59300848
##  [6,]  0.23741743 -0.28994021
##  [7,]  0.44590987 -0.16947415
##  [8,]  0.02359128  0.23749672
##  [9,] -0.38360006  0.01647714
## [10,] -0.06548292 -0.05685288
```

```
colMeans(results_cca_cv)
```

```
##         CV1         CV2
##  0.07605609 -0.07763294
```

Visualize out of sample cannonical correlations

```
plot(cbind(results_cca_cv[,1], results_cca_cv[,2]), 1:20,
     yaxt="n",
     xlim=c(-1, 1),
     bty='n',
     ylab='Cross-validation fold',
     xlab='Test-set correlation',
     main='Out of sample correlation',
     pch=c(rep(19,10), rep(1,10)))

axis(2, at=c(1:20), labels=c(10:1, 10:1))#, lty='blank')
```

```
abline(v=0, col='grey')
legend("topleft", c('CV1', 'CV2'), bty='n', pch=c(19,1))
```

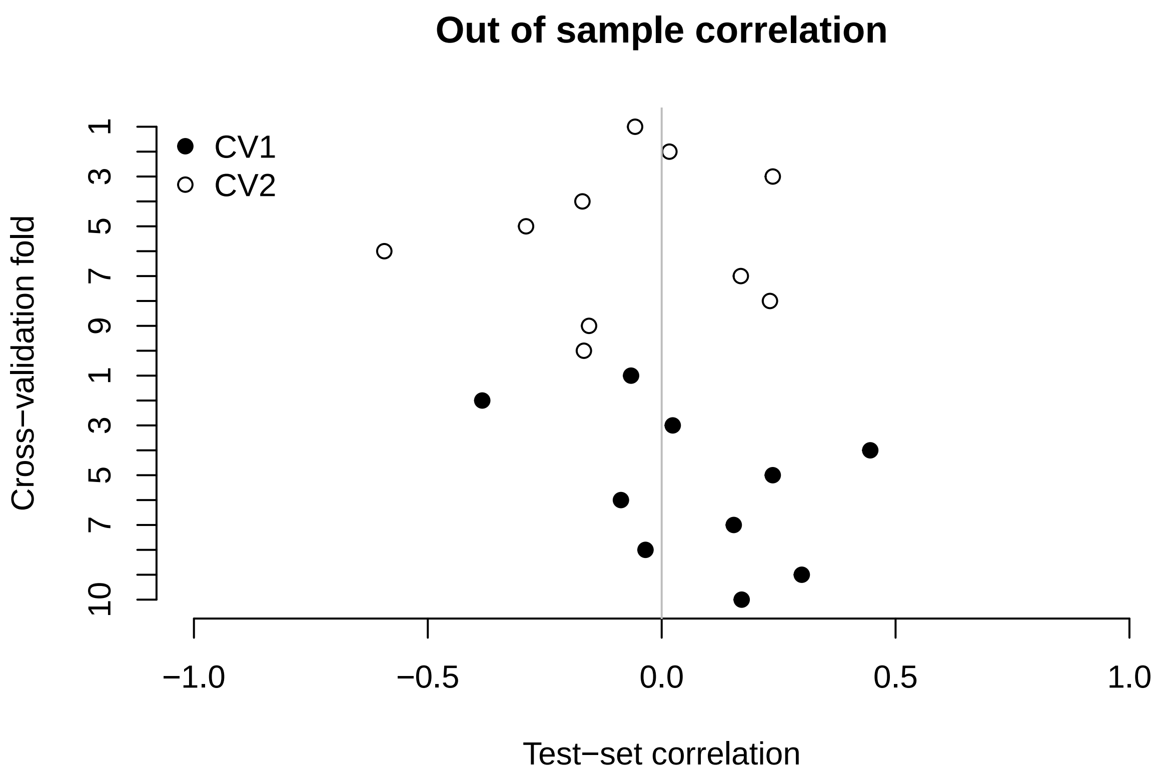

Stability of canonical loadings

Create function that performes leave-one-out jackknife procedure to get uncertainity of canonical loadings taking into an account uncertainity caused by feature selection.

Jackknife repeatedly leaves one subject out and then performs the feature selection and CCA procedure in the same way as above.

```
njack <- nrow(residual_rs_connectivity)
jack_res <- foreach(i=1:njack) %dopar% {
  model <- select_and_cca_fit(residual_rs_connectivity[-i,],
                              clinical[-i,],
                              n_selected_vars)
  selected.vars <- model$names$X
  prediction <- predict.cancor(model,
                              residual_rs_connectivity[i, selected.vars],
                              clinical[i,])
  list(prediction, model)
}
```

run jackknife

```
jack.results <- lapply(jack_res, function(x){return(x[[1]])})
jack.X <- lapply(jack.results, function(x){return(x[[1]])})
jack.X <- as.data.frame(do.call(rbind, jack.X))
jack.Y <- lapply(jack.results, function(x){return(x[[2]])})
jack.Y <- as.data.frame(do.call(rbind, jack.Y))
```

get loadings from saved jackknife models

```
jack_models <- lapply(jack_res, function(x){return(x[[2]])})
jack.loadings <- lapply(jack_models, function(model){
  return(model$structure$Y.yscores[,1])})
jack.loadings <- as.data.frame(do.call(rbind, jack.loadings))
```

plot distribution of canonical loadings across all jackknife models

```
library(reshape2)
melted.loadings <- melt(jack.loadings)

par(mfrow=c(1,2), las=1, mai=c(1.02, 1.3, 0.82, 0.42))
boxplot(abs(value) ~ variable, data=melted.loadings, horizontal=T)
plot(abs(melted.loadings$value),
     jitter(as.numeric(melted.loadings$variable)),
     pch='.')
```

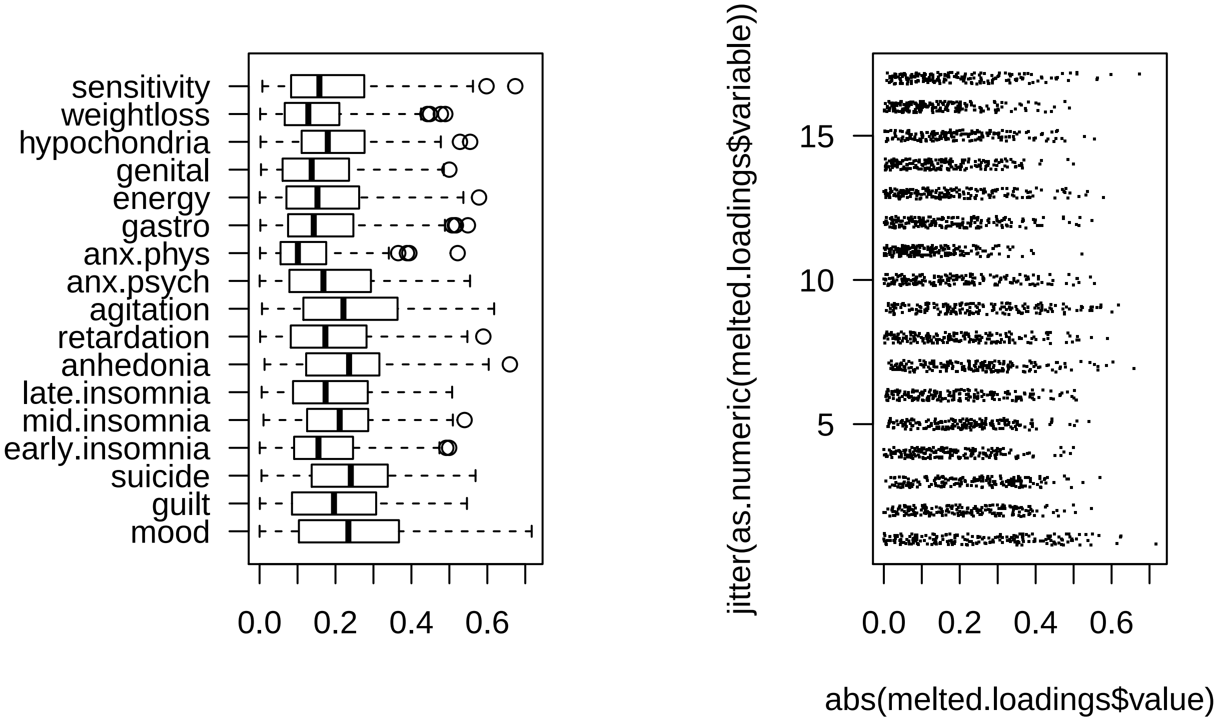

Compare loadings with original study

Canonical loadings as presented in original study

```
Drysdale_1 <- c( 0, 0.41, 0.32, 0.59, 0.54, 0, 0, 0, 0, 0,
                 0.65, 0, 0, 0.24, 0.25, 0, 0)
Drysdale_2 <- c(0.27, 0.25, 0.26, 0, 0, 0, 0.83, 0.36, 0.23,
                0, 0, 0, -0.35, 0, 0.21, 0, 0)
```

Plot loadings obtained above together with loadings from the original study

```
dr_c <- rbind(Drysdale_1, Drysdale_2)

new_cors <- rbind(t(cca_y_loadings), dr_c)
new_cors_thr <- new_cors
new_cors_thr[abs(new_cors) < 0.2] = 0

library(corrplot)
```

```
## corrplot 0.84 loaded
```

```
corrplot(new_cors, method = 'color', cl.ratio=0.1, cl.align = 'l',
         addCoef.col = "black", tl.srt = 45,number.cex = .5,
         col=colorRampPalette(c("blue", "white", "red"))(200))
```

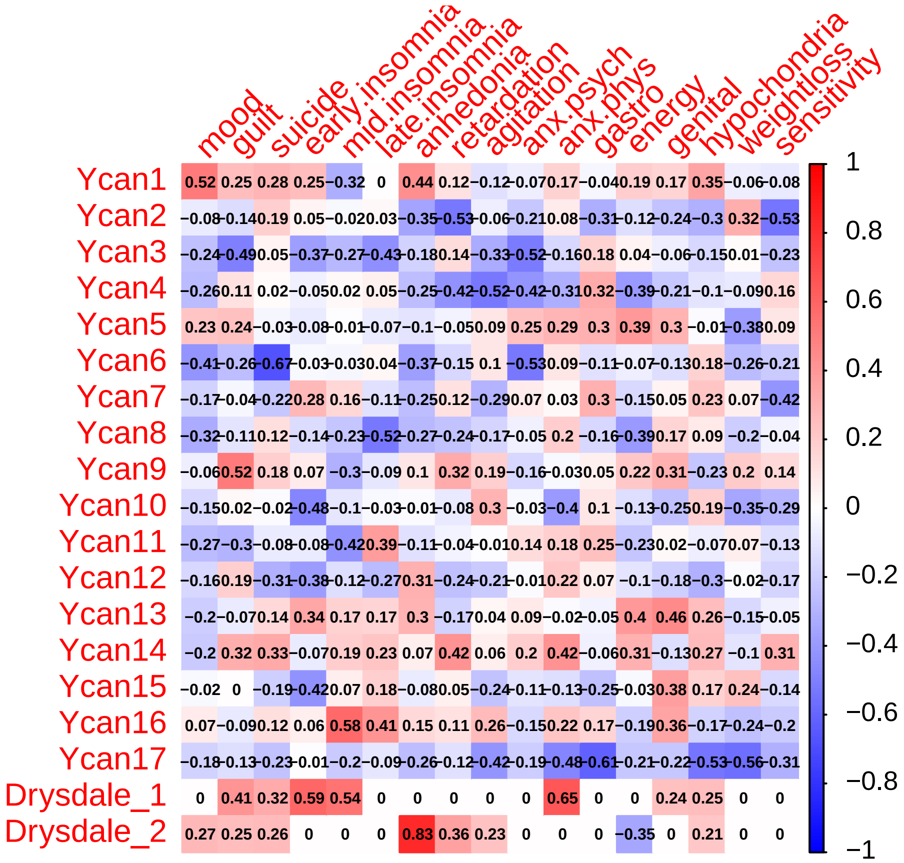

2. Clustering analysis

Run hierarchical clustering

Plot subjects based on their first 2 RS canonical variates values and their 4 cluster solution as in the original study

```
par(mfrow=c(1,2))
cca_rs_data <- canonical.variates$X_pred[,1:2]
plot(cca_rs_data,
     xlab = "RS connectivity variate 1", ylab = "RS connectivity variate 2")

library(stats)
d <- dist(cca_rs_data, method = "euclidean")
res.hc <- hclust(d, method = "ward.D" )
clusters <- cutree(res.hc, k = 4)
plot(cca_rs_data,
     xlab = "RS connectivity variate 1", ylab = "RS connectivity variate 2",
     col=clusters)
```

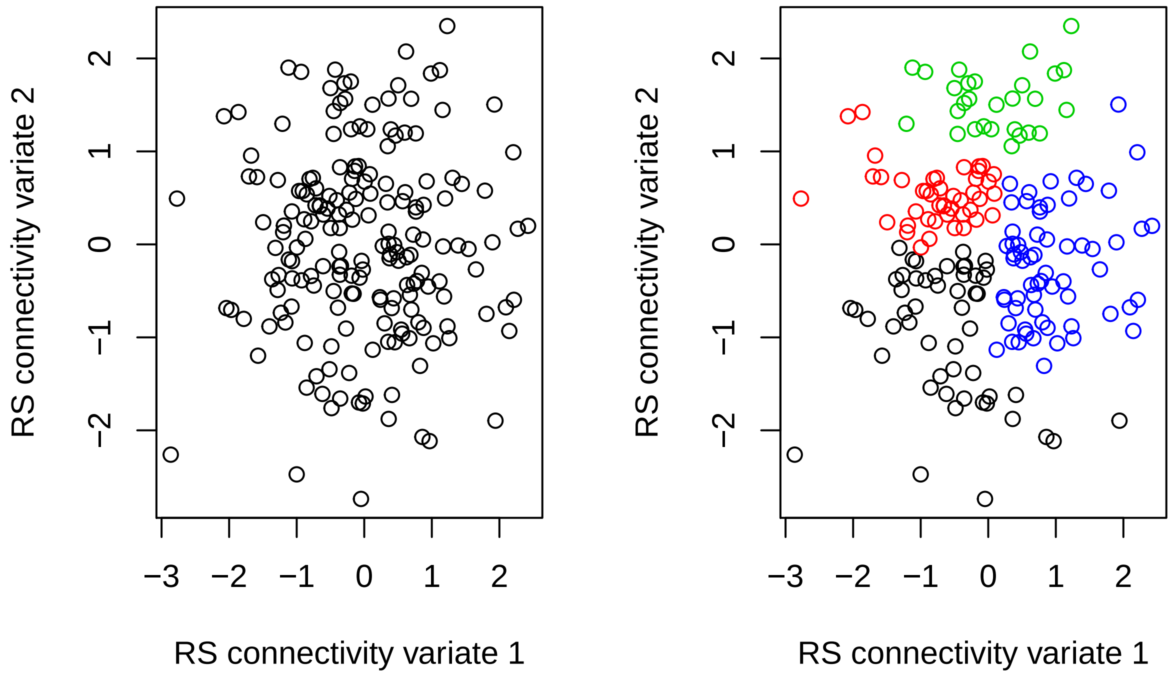

Stability of cluster assignment

We will make the same plot as above but we will use canonical variates from one of jackknife models estimated before (by leaving one subject out) but using colors according to previous cluster assignment. Thus showing how relative positions of subjects change with respect to small perturbation of the data.

```
jack.pred <- lapply(jack_models, function(model){
                        predict.cancor(model,
                                       residual_rs_connectivity[,model$names$X],
                                       clinical)})
par(mfrow=c(1,2))
plot(cca_rs_data, col=clusters,
     xlab = "RS connectivity variate 1",
     ylab = "RS connectivity variate 2")
plot((-1)*jack.pred[[1]]$X_pred[,1], jack.pred[[1]]$X_pred[,2], col=clusters,
     xlab = "refited RS connectivity variate 1",
     ylab = "refited RS connectivity variate 2")
```

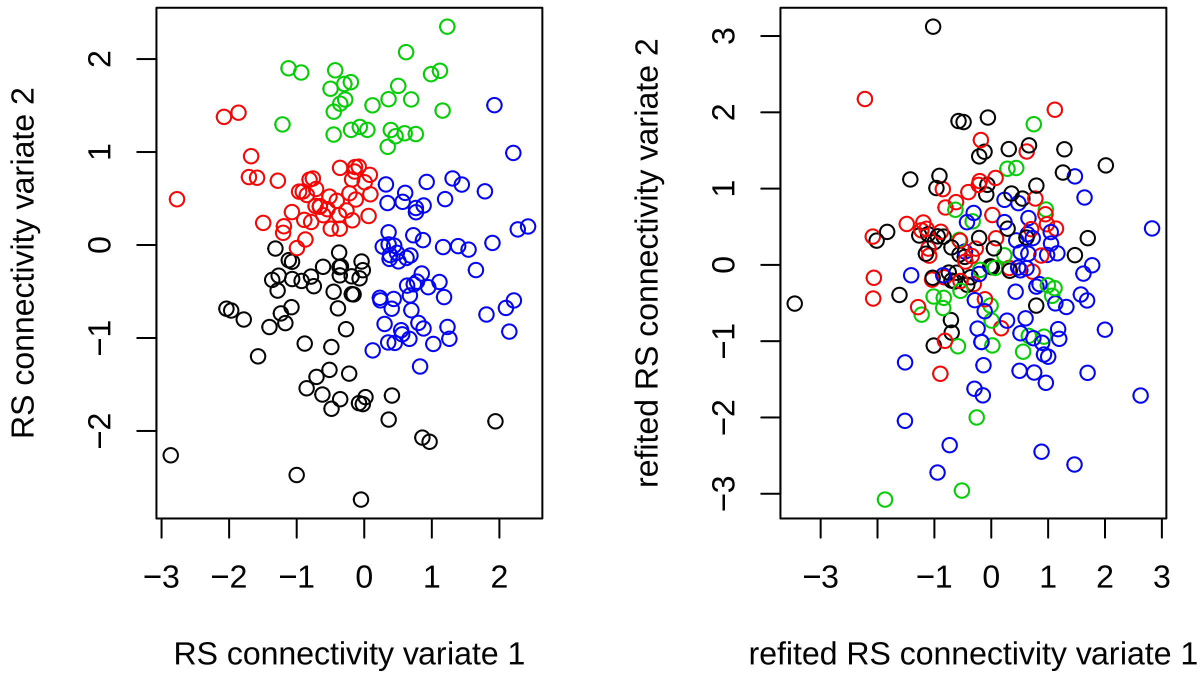

Clustering indeces

Compute and plot clustering indexes

```
library(NbClust)
hcfit_ch <- NbClust(cca_rs_data, method="ward.D",
                    min.nc = 2, max.nc = 6, index = "ch")
hcfit_sl <- NbClust(cca_rs_data, method="ward.D",
                    min.nc = 2, max.nc = 6, index = "silhouette")

par(mfrow=c(1,2))
plot(names(hcfit_ch$All.index), hcfit_ch$All.index,
     main = "variance ratio criterion\n (Calinski-Harabasz index)",
```

```
    xlab = "Number of clusters", ylab="variance ratio criterion", type='b')
plot(names(hcfit_sl$All.index), hcfit_sl$All.index,
     main = "Silhoutte", xlab = "Number of clusters", ylab="Silhoutte",
     type='b')
```

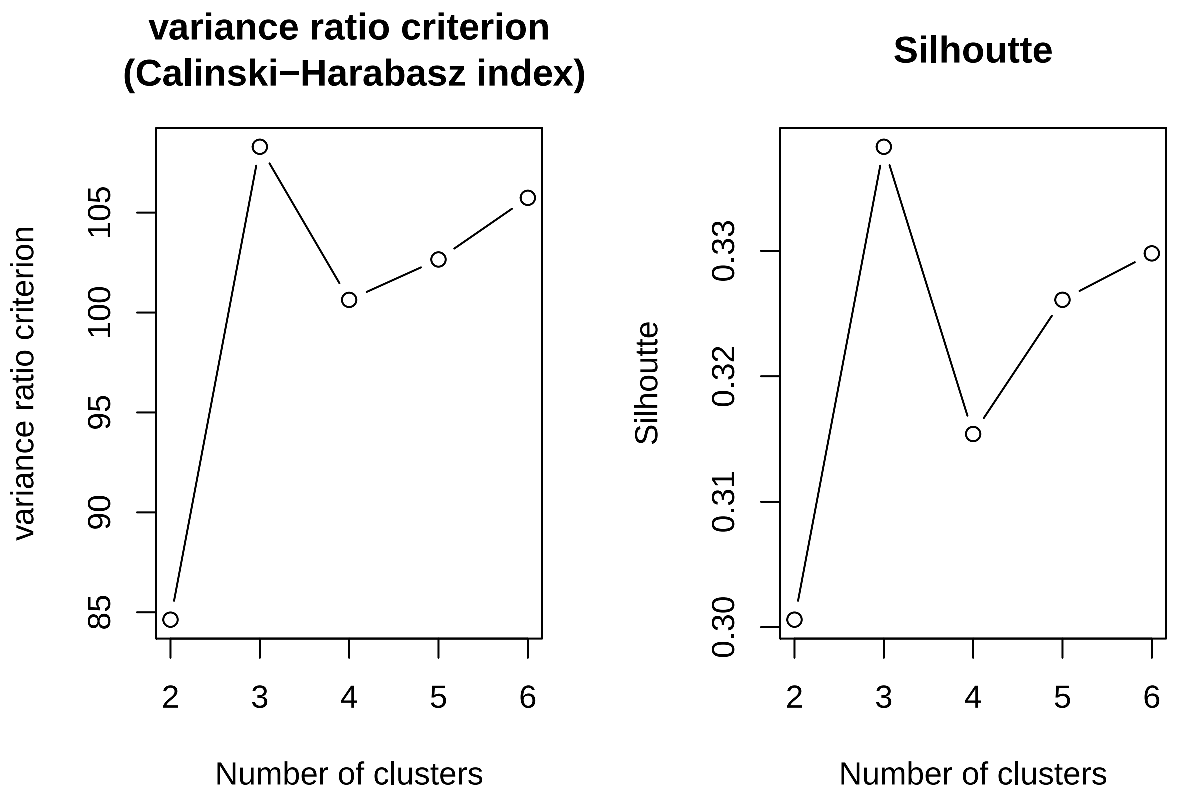

Statisticall significance of clusters

Make a function that performs a hierarchical clustering and return the highest clustering indexes

```
cluster_test <- function(cca_data){
  #ugly hack, because i don't know how to prevent this library creating many plots
  hcfit <- NbClust(cca_data, method="ward.D", index="ch", min.nc=3, max.nc = 5)
  CH_index <- max(hcfit$All.index)
  hcfit <- NbClust(cca_data, method="ward.D", index="silhouette", min.nc=3, max.nc = 5)
  sil_index <- max(hcfit$All.index)
  return(c("CH"=CH_index, "Silhouette"=sil_index))
}
```

Fit a multivariate normal distribution to the same data used to perform hierarchical clustering

```
library(MASS)
sigma <- cov(cca_rs_data)
mu <- colMeans(cca_rs_data)
real_CI <- cluster_test(cca_rs_data)
```

Repeatedly perform hierarchical clustering on samples from this distribution, thus creating an empirical null

distribution of clustering indeces

```
# get a null distribution of clusters
null_CI <- list()
n_sims <- 1999
for (i in 1:n_sims){
  rand_sample <- mvrnorm(n=nrow(cca_rs_data), mu=mu, Sigma=sigma)
  null_CI[[i]] <- cluster_test(rand_sample)
}
null_CI <- as.data.frame(do.call(rbind, null_CI))
```

print p-values

```
rank_cv1 <- sum(real_CI[1] < null_CI[,1]) + 1
pval_cv1 <- rank_cv1 / (n_sims+1)
rank_cv2 <- sum(real_CI[2] < null_CI[,2]) + 1
pval_cv2 <- rank_cv2 / (n_sims+1)
t(t((c("p.val variance ratio"=pval_cv1, "p.val Silhouette"=pval_cv2))))
```

```
##                          [,1]
## p.val variance ratio 0.4465
## p.val Silhouette     0.1940
```

visualize null distribution

```
par(mfrow=c(1,2))
hist(null_CI[,1], breaks = 30, main = "variance ratio criterion null")
abline(v=real_CI[1], col="red")
text(real_CI[1] + 10, 70, paste('p = ', round(pval_cv1, 2)))

hist(null_CI[,2], breaks = 30, main = "Silhouette null")
abline(v=real_CI[2], col="red")
text(real_CI[2] - 0.025, 80, paste('p = ', round(pval_cv2, 2)))
```

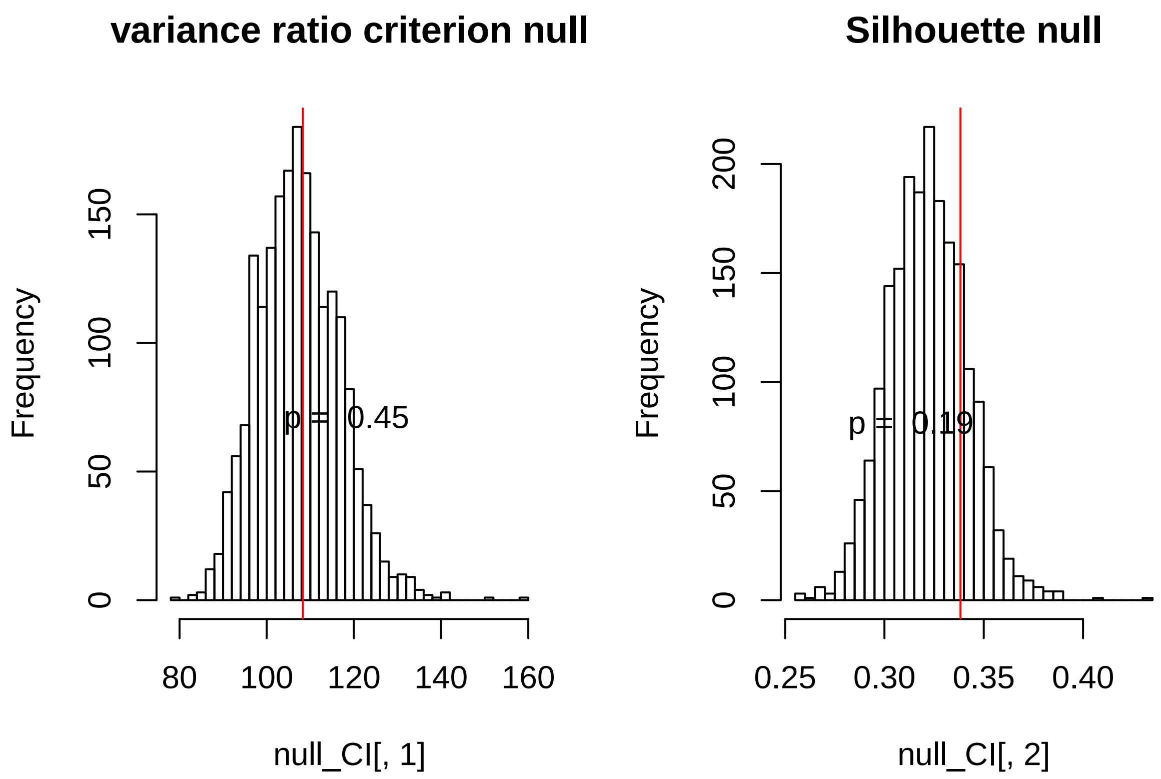

3. Software enviroment

```r
sessionInfo()
```

```
## R version 3.5.2 (2018-12-20)
## Platform: x86_64-pc-linux-gnu (64-bit)
## Running under: Linux Mint 18
##
## Matrix products: default
## BLAS: /usr/lib/libblas/libblas.so.3.6.0
## LAPACK: /usr/lib/lapack/liblapack.so.3.6.0
##
## locale:
##  [1] LC_CTYPE=en_US.UTF-8       LC_NUMERIC=C
##  [3] LC_TIME=en_US.UTF-8        LC_COLLATE=en_US.UTF-8
##  [5] LC_MONETARY=nl_NL.UTF-8    LC_MESSAGES=en_US.UTF-8
##  [7] LC_PAPER=nl_NL.UTF-8       LC_NAME=C
##  [9] LC_ADDRESS=C               LC_TELEPHONE=C
## [11] LC_MEASUREMENT=nl_NL.UTF-8 LC_IDENTIFICATION=C
##
## attached base packages:
## [1] parallel  stats     graphics  grDevices utils     datasets  methods
## [8] base
##
```

```
## other attached packages:
##  [1] MASS_7.3-51.1     NbClust_3.0       corrplot_0.84
##  [4] reshape2_1.4.3    doMC_1.3.5        iterators_1.0.10
##  [7] foreach_1.4.4     permute_0.9-5     candisc_0.8-0
## [10] heplots_1.3-5     car_3.0-2         carData_3.0-2
## [13] caret_6.0-81      ggplot2_3.1.0     lattice_0.20-38
## [16] psych_1.8.12      data.table_1.12.0
##
## loaded via a namespace (and not attached):
##  [1] Rcpp_1.0.0          lubridate_1.7.4    class_7.3-15
##  [4] assertthat_0.2.0   digest_0.6.18      ipred_0.9-8
##  [7] R6_2.4.0            cellranger_1.1.0   plyr_1.8.4
## [10] stats4_3.5.2       evaluate_0.13      pillar_1.3.1
## [13] rlang_0.3.1        lazyeval_0.2.1     curl_3.3
## [16] readxl_1.3.0       rpart_4.1-13       Matrix_1.2-15
## [19] rmarkdown_1.11     splines_3.5.2      gower_0.1.2
## [22] stringr_1.4.0      foreign_0.8-71     munsell_0.5.0
## [25] compiler_3.5.2     xfun_0.5           pkgconfig_2.0.2
## [28] mnormt_1.5-5       htmltools_0.3.6    nnet_7.3-12
## [31] tidyselect_0.2.5   tibble_2.0.1       prodlim_2018.04.18
## [34] rio_0.5.16         codetools_0.2-16   crayon_1.3.4
## [37] dplyr_0.8.0.1      withr_2.1.2        recipes_0.1.4
## [40] ModelMetrics_1.2.2 grid_3.5.2         nlme_3.1-137
## [43] gtable_0.2.0       magrittr_1.5       scales_1.0.0
## [46] zip_2.0.0          stringi_1.3.1      timeDate_3043.102
## [49] generics_0.0.2     openxlsx_4.1.0     lava_1.6.5
## [52] tools_3.5.2        forcats_0.4.0      glue_1.3.0
## [55] purrr_0.3.1        hms_0.4.2          abind_1.4-5
## [58] survival_2.43-3    yaml_2.2.0         colorspace_1.4-0
## [61] knitr_1.21         haven_2.1.0
```
